# Neonatal neuronal WWOX gene therapy rescues *Wwox* null phenotypes

**DOI:** 10.15252/emmm.202114599

**Published:** 2021-11-07

**Authors:** Srinivasarao Repudi, Irina Kustanovich, Sara Abu‐Swai, Shani Stern, Rami I Aqeilan

**Affiliations:** ^1^ The Concern Foundation Laboratories, The Lautenberg Center for Immunology and Cancer Research, Immunology and Cancer Research‐IMRIC Hebrew University‐Hadassah Medical School Jerusalem Israel; ^2^ Sagol Department of Neurobiology University of Haifa Haifa Israel

**Keywords:** AAV9, DEE28, hypomyelination, seizures, WOREE syndrome, Biotechnology & Synthetic Biology, Genetics, Gene Therapy & Genetic Disease, Neuroscience

## Abstract

WW domain‐containing oxidoreductase (*WWOX*) is an emerging neural gene‐regulating homeostasis of the central nervous system. Germline biallelic mutations in *WWOX* cause WWOX‐related epileptic encephalopathy (WOREE) syndrome and spinocerebellar ataxia and autosomal recessive 12 (SCAR12), two devastating neurodevelopmental disorders with highly heterogenous clinical outcomes, the most common being severe epileptic encephalopathy and profound global developmental delay. We recently demonstrated that neuronal ablation of murine *Wwox* recapitulates phenotypes of *Wwox*‐null mice leading to intractable epilepsy, hypomyelination, and postnatal lethality. Here, we designed and produced an adeno‐associated viral vector (AAV9) harboring murine *Wwox* or human *WWOX* cDNA and driven by the human neuronal Synapsin I promoter (*AAV‐SynI‐WWOX*). Testing the efficacy of AAV‐SynI‐WWOX delivery in *Wwox*‐null mice demonstrated that specific neuronal restoration of WWOX expression rescued brain hyperexcitability and seizures, hypoglycemia, myelination deficits, and the premature lethality and behavioral deficits of *Wwox*‐null mice. These findings provide a proof‐of‐concept for *WWOX* gene therapy as a promising approach to curing children with WOREE and SCAR12.

## Graphical abstract

AAV‐mediated neuronal WWOX restoration reverses abnormal defects of *Wwox* null mice, providing a proof‐of‐concept for *WWOX* gene therapy as a promising approach for treating children with WOREE syndrome.

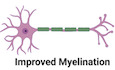

**The paper explained**

**Problem**

Germline mutations in WW domain‐containing oxidoreductase (*WWOX)* result in loss of function of WWOX and cause severe developmental and epileptic encephalopathy (DEE) known as WWOX‐related epileptic encephalopathies (WOREE syndrome) and Spinocerebellar ataxia type 12 (SCAR12 syndrome). Response to antiepileptic drugs of these syndromes is very poor, and hence, alternative treatment approaches should be explored and developed.

**Results**

In this study, we used an adeno‐associated virus (AAV) vector to restore WWOX expression in the brain of *Wwox*‐null mice, which recapitulates many of the phenotypes of WOREE syndrome. We demonstrated that a single intracerebroventricular injection of AAV9‐Synapsin I‐WWOX could rescue the growth retardation, hypoglycemia, epileptic seizures, ataxia, and premature death of *Wwox*‐null mice. In addition, WWOX restoration improved myelination and reversed the abnormal behavioral changes of *Wwox*‐null mice.

**Impact**

These remarkable results indicate that WWOX gene therapy could be a promising approach to cure children with WOREE and SCAR12 syndromes.

## Introduction

The WW domain‐containing oxidoreductase (*WWOX*) gene maps to chromosome 16q23.1‐q23.2 encompassing the chromosomal fragile site FRA16D and encodes a 46‐kDa WWOX protein (Del Mare *et al*, 2009; Aldaz *et al*, 2014). WWOX comprises two WW domains (WW1 and WW2) and an extended short‐chain dehydrogenase/reductase (SDR) domain (Chang *et al*, 2007; Salah *et al*, 2010, 2012). Through its WW1 domain, WWOX physically interacts with several key signaling proteins (DVL, AP‐2, ErbB‐4, HIF1‐α, p53, p63, p73, c‐JUN, ITCH, and RUNX2) and suppresses tumor progression in several cancer cell types (Abu‐Remaileh *et al*, 2015). Additionally, WWOX has been shown to regulate DNA damage response, glucose homeostasis, cell metabolism, and neuronal differentiation (Aqeilan *et al*, 2014; Hazan *et al*, 2016).

In recent years, evidence linking WWOX function to the regulation of homeostasis of the central nervous system (CNS) has been proposed (Aldaz & Hussain, 2020; Banne *et al*, 2021). Germline recessive mutations (missense, nonsense, and partial/complete deletions) in the *WWOX* gene were found to be associated with two major phenotypes, namely SCAR12 (spinocerebellar ataxia, autosomal recessive 12, OMIM 614322) and WOREE syndrome (WWOX‐related epileptic encephalopathy), the latter also known as developmental and epileptic encephalopathy 28 (DEE28, OMIM 616211) (Banne *et al*, 2021). WOREE is a complex and devastating neurological disorder observed in children harboring an early premature stop codon or mutations that result in loss of WWOX function (Piard *et al*, 2019). The clinical spectrum of WOREE syndrome includes severe developmental delay, early onset of severe epilepsy with variable seizure manifestations (tonic, clonic, tonic–clonic, myoclonic, infantile spasms, and absence). Most of the affected patients make no eye contact and are not able to sit, speak, or walk (Banne *et al*, 2021). WOREE syndrome is refractory to current antiepileptic drugs (AEDs); hence, there is an urgent need to develop alternative treatments to help children with WOREE syndrome. Children with SCAR12, mostly due to missense mutations in *WWOX*, display a milder phenotype including ataxia and epilepsy (Mallaret *et al*, 2014). Epilepsy in SCAR12 can be treated with available AEDs, though children still display ataxia and are intellectually disabled. Moreover, WWOX mutations have been documented in patients with West syndrome, which is characterized by epileptic spasms with hypsarrhythmia (Shaukat *et al*, 2018). Brains of the children carrying *WWOX* gene mutations are found to be abnormal, as assessed by magnetic resonance imaging (MRI). Brain abnormalities such as hypoplasia of the corpus callosum, progressive cerebral atrophy, delayed myelination, and optic nerve atrophy have been documented in most cases (Banne *et al*, 2021). It is largely unknown how mutations in *WWOX* or loss of WWOX’s function could lead to these CNS‐associated abnormalities.

There is a marked similarity between human *WWOX* (*hWWOX*) and murine *Wwox* (*mWwox*) sequence (Richards *et al*, 2015). In fact, the human WWOX protein is 93% identical and 95% similar to the murine WWOX protein sequence. Remarkably, targeted loss of *Wwox* function in rodent models (mice and rats) phenocopies the complex human neurological phenotypes, including severe epileptic seizures, growth retardation, ataxia, and premature death (Suzuki *et al*, 2009; Mallaret *et al*, 2014; Tanna & Aqeilan, 2018). *Wwox*‐null mice also exhibit phenotypes associated with hypoglycemia, impaired bone metabolism, and steroidogenesis (Aqeilan *et al*, 2008, 2009; Abu‐Remaileh & Aqeilan, 2014).

In a recent study, we found that conditional ablation of murine *Wwox* in either neural stem cells and progenitors (N‐KO) or neuronal cells (S‐KO mice) resulted in severe epilepsy, ataxia, and premature death at 3–4 weeks, recapitulating the phenotypes observed in the *Wwox*‐null mice (Repudi *et al*, 2021). These results highlight the significant role of WWOX in neuronal function and prompted us to test whether restoring WWOX expression in the neuronal compartment of *Wwox*‐null mice could reverse the observed phenotypes. To this end, we used an adeno‐associated virus (AAV) vector to restore WWOX expression. AAV is a promising candidate for gene therapy in many disorders including neuromuscular, CNS, and ocular disorders (Pierce & Bennett, 2015; Aguti *et al*, 2018; Sun & Schaffer, 2018; Wang *et al*, 2019). Moreover, AAV appears to elicit little to no immune response and integrates into the host at very low rates, which reduces the risks of genotoxicity (Nakai *et al*, 2001). In our current study, we demonstrated that an AAV vector harboring the *mWwox* or *hWWOX* open reading frame and driven by the human neuronal Synapsin I promoter could reverse *Wwox* null phenotypes. A single intracerebroventricular (ICV) injection of AAV9‐Synapsin I‐WWOX rescued the growth retardation, hypoglycemia, epileptic seizures, ataxia, and premature death of *Wwox*‐null mice. In addition, WWOX restoration improved myelination and reversed the abnormal behavioral changes of *Wwox*‐null mice. Overall, these remarkable results indicate that WWOX gene therapy could be a promising cure approach for children with WOREE and SCAR12.

## Results

### Restoration of neuronal WWOX rescues growth retardation and post‐natal lethality

In a recent study, we reported that conditional ablation of WWOX in neurons phenocopies the *Wwox*‐null mice including growth retardation, spontaneous epileptic seizures, ataxia, and premature death at 3–4 weeks (Repudi *et al*, 2021). These results implied that WWOX is a key neuronal gene‐regulating homeostasis of the CNS. Prompted by these remarkable findings, we set to address whether neuronal‐specific expression of WWOX in *Wwox*‐null mice could rescue lethality of these mice and their associated phenotypes. We designed an adeno‐associated viral (AAV) vector to express m*Wwox* or h*WWOX* cDNA driven by a human Synapsin‐I (hSynI) promoter (Appendix Fig S1A) and packaged them into an AAV9 serotype, which has high CNS tropism and has been used in CNS‐based gene therapy trials (Balakrishnan & Jayandharan, 2014; Wang *et al*, 2019). An *IRES‐EGFP* sequence was cloned downstream of the m*Wwox* sequence to allow tracking of expression. Successful delivery of AAV9‐hSynI‐mWwox‐IRES‐EGFP (AAV‐mWwox) or AAV9‐hSynI‐hWWOX (AAV‐hWWOX) should lead to expression of intact WWOX protein in Synapsin‐I‐positive non‐dividing/matured neurons. As control, AAV9‐hSynI‐EGFP was used. Expression of WWOX and GFP was initially validated by infecting primary *Wwox*‐null dorsal root ganglion (DRGs) neurons with the viral particles *in vitro* (Appendix Fig S1B).

**Figure 1:**
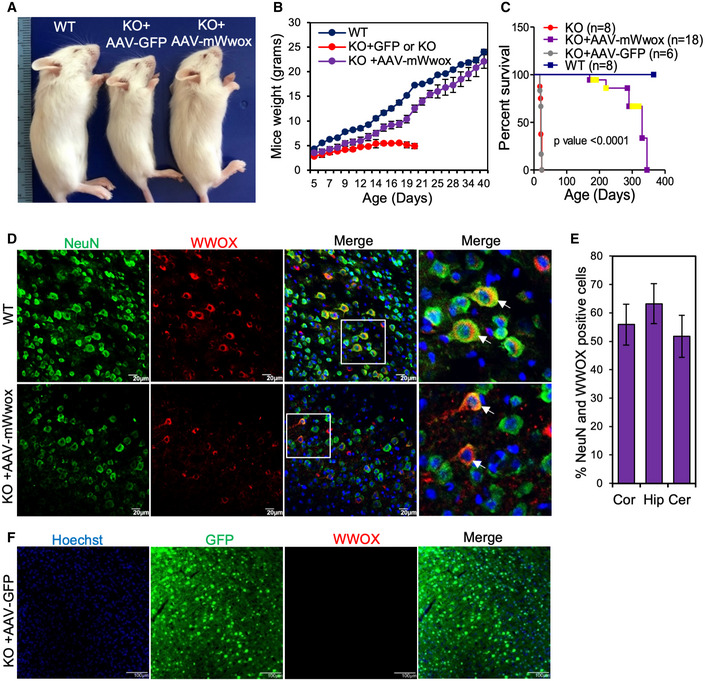
Restoration of mWwox in neurons improves growth of *Wwox*‐null mice and extends their life span Physical appearance of wild‐type (WT), *Wwox* null injected either with AAV9‐hSynI‐GFP (the control virus), or AAV9‐hSynI‐mWwox‐IRES‐GFP virus at P17.Graph showing the body weight of WT, KO, and KO+ AAV‐mWwox at different days after birth (*n* = 4 per group).Kaplan–Meier survival graph indicates prolonged life span of *Wwox* knockout mice injected with AAV9‐hSynI‐mWwox [total *n* = 18, spontaneously dead *n* = 6, mice taken out for electrophysiology/electron microscopy/analysis, are shown in yellow, *n* = 12] compared to mice injected with AAV9‐hSynI‐GFP (*n* = 6) or the non‐injected (*n* = 8); *P* < 0.0001, log‐rank Mantel–Cox test.Immunofluorescence images showing the expression of WWOX (red) in brain tissue (cortex) at P17 from WT and *Wwox*‐null mice that were injected with AAV9‐hSynI‐mWwox (2 × 10^10^). Neurons are labeled with anti‐NeuN antibody (green). The square white box shows the magnified area. White arrows show the transgene expression in NeuN‐positive cells.Graph showing the percentage of NeuN and WWOX double‐positive cells in different parts of the brain (cortex, hippocampus, and cerebellum) from KO mice injected with AAV‐mWwox at P17. NeuN and WWOX double‐positive cells were calculated from 3 identical sagittal sections of the AAV‐mWwox‐injected mice (*n* = 3) brains.Expression of EGFP (green) is shown in brain tissue (cortex) of *Wwox* null injected with AAV9‐hSynI‐GFP (2 × 10^10^). Data information: Error bars represent ± SD. Scale bars D) 20 µm, F) 100 µm.

**Figure 2:**
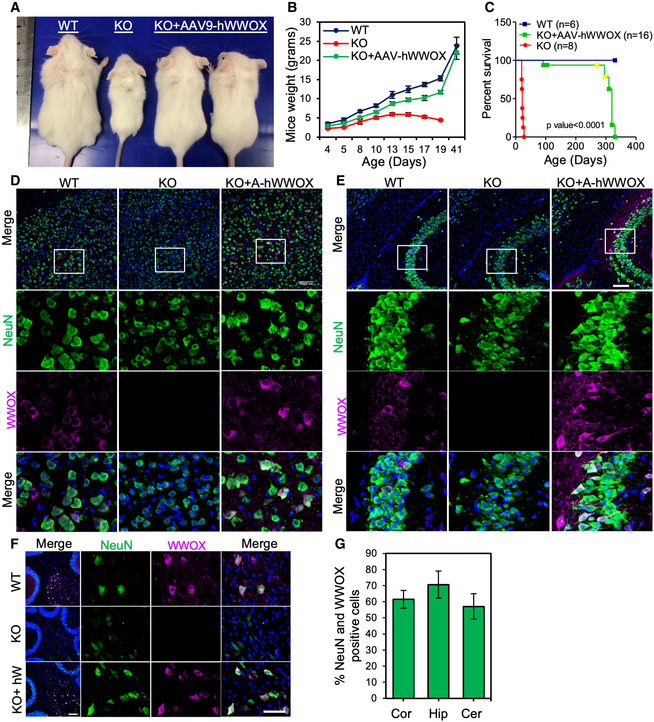
Restoration of hWWOX in neurons reverse the phenotypes of *Wwox*‐null mice and extends their life span APhysical appearance of wild type (WT), *Wwox* null (KO), or KO injected with AAV‐hWWOX (2 × 10^10^) at P19.BGraph showing body weight of WT, KO, and KO+AAV‐hWWOX at different days after birth. *n* = 3 mice per genotype.CKaplan–Meier survival graph indicates prolonged life span of *Wwox* knockout mice injected with AAV9‐hWWOX [total *n* = 16, alive *n* = 6, spontaneously dead *n* = 6, 4 mice (shown in yellow) were taken out for analysis) compared to the non‐injected (*n* = 8)] (*P* < 0.0001, log‐rank Mantel–Cox test).D–FImmunofluorescence images showing the expression of WWOX (magenta) in cortex (D), hippocampus (E), and cerebellum (F) at P19 from WT and *Wwox*‐null mice that were injected with AAV‐hWWOX (2 × 10^10^) or *Wwox* null alone. Neurons are labeled with anti‐NeuN antibody (green). The square white box shows the magnified area.GGraph represents the percentage of neurons showing the transgene (NeuN and WWOX positive) expression at different parts (cortex, hippocampus, and cerebellum) of the KO brain at P19 after AAV injection. NeuN and WWOX double‐positive cells were calculated from 3 identical sagittal sections of the AAV‐hWWOX‐injected mice (*n* = 3) brains. Data information: Error bars represent ± SD. Scale bars D), E) 100 µm, F) 50 µm.

We then evaluated the expression and function of the AAV constructs *in vivo*. Viral particles (2 × 10^10^/hemisphere) of AAV9‐hSynI‐mWwox, AAV9‐hSynI‐hWWOX, or AAV9‐hSynI‐EGFP were injected into the ICV region of *Wwox*‐null mice at birth (P0), to achieve widespread transduction of neurons throughout the brain (Gholizadeh *et al*, 2013; Kim *et al*, 2014). Monitoring of the treated mice revealed that mice injected with either AAV9‐hSynI‐mWwox (Fig 1A) or AAV9‐hSynI‐hWWOX (Fig 2A) grew normally, gradually gained weight, and were indistinguishable from wild type by the age of 6 weeks (Figs 1B and 2B). AAV9‐hSynI‐EGFP‐injected mice exhibited similar phenotypes of *Wwox*‐null mice (Fig 1B). Of note, *Wwox*‐null and AAV9‐hSynI‐EGFP‐injected mice were hypoglycemic from the second week until they succumbed (Aqeilan *et al*, 2008; Abu‐Remaileh & Aqeilan, 2014), while AAV9‐hSynI‐mWwox‐ or AAV9‐hSynI‐hWWOX‐injected mice had normal blood glucose levels when compared to the wild‐type mice (Appendix Fig S1C and D). Remarkably, all rescued mice, treated with AAV‐mWwox or AAV‐hWWOX, exhibited extended life span compared to the *Wwox*‐null or AAV9‐hSynI‐EGFP‐injected mice (Figs 1C and 2C). No difference was noted when using the murine or human WWOX vectors. These findings imply that neuronal WWOX restoration could have the potential to rescue the lethality of *Wwox*‐null mice.

### Specific neuronal WWOX expression upon use of AAV9‐SynI‐WWOX vectors

Promoted by the above‐observed outcomes, we next set to validate specific neuronal expression of the transgene (mWwox or hWWOX) in different brain regions. To this end, we performed immunofluorescence staining using anti‐NeuN and anti‐WWOX antibodies in the injected mice. Specific neuronal WWOX expression was detected in cortex (Figs 1D and 2D), hippocampus (Fig 2E), and cerebellum (Fig 2F) of P17‐P19‐treated mice. The percentage of NeuN and WWOX double‐positive cells was calculated and found to range between 60 and 70% (Figs 1E and 2G). Furthermore, WWOX protein expression was lasting as it was detected in 9 months post‐injection (Appendix Fig S2A). The expression of GFP was detected in KO injected with AAV9‐GFP (Fig 1F). Importantly, WWOX expression was lacking in non‐neuronal cells of the brain such as oligodendrocytes of KO mice injected with either AAV‐mWwox (Appendix Fig S1E) or AAV‐hWWOX (Appendix Fig S1F), as assessed by co‐staining with CC1 and anti‐WWOX antibodies. These results further confirm that the restoration of WWOX expression is specific to the neuronal compartment.

To rule out leakiness and non‐specific expression of AAV9‐WWOX, we also tested expression of the transgene in peripheral tissues, including liver, pancreas, kidney, testis, and ovary of juvenile and aged mice. As presented in Appendix Fig S2B and C, no WWOX expression was detected in *Wwox*‐null tissues in P17 and 9‐month‐old rescued mice. Altogether, these results reveal that specific neuronal WWOX expression of AAV9‐SynI‐WWOX vectors is responsible for rescuing *Wwox*‐null mice phenotypes.

### Effect of neuronal WWOX restoration on ataxia, fertility, and bone phenotypes of *Wwox*‐null mutant mice

We next set to test the effect of WWOX neuronal restoration on other previously reported phenotypes of *Wwox*‐null mice. As WWOX loss of function was previously shown to exhibit impaired motor coordination and ataxic phenotype, we next tested whether WWOX restoration could reverse this phenotype. Remarkably, WWOX single ICV injection improved motor coordination in rescued mice as presented by hindlimb clasping test (Appendix Fig S3). Furthermore, the rescued mice were active and both males and females were fertile. Moreover, since *Wwox*‐null mice were previously shown to lack testicular Leydig cells (Aqeilan *et al*, 2009), we next determined how WWOX neuronal restoration affected this phenotype and found intact Leydig cells in P17 AAV9‐hSynI‐WWOX‐treated mice (Appendix Fig S4A). Bone growth defects were also formerly documented in *Wwox* mutant mice (Aqeilan *et al*, 2008; Ludes‐Meyers *et al*, 2009; Kurek *et al*, 2010; Abdeen, Del Mare, *et al*, 2013; Del Mare *et al*, 2016), and hence, we examined bones of rescued mice and observed that cortical bones were of comparable size and thickness to WT mice (Appendix Fig S4B). Altogether, our results indicate that WWOX neuronal restoration has a wide‐reaching effect on *Wwox*‐null mice phenotypes.

### Neuronal restoration of WWOX decreases hyperexcitability and neuroinflammation in *Wwox*‐null mice

**Figure 3:**
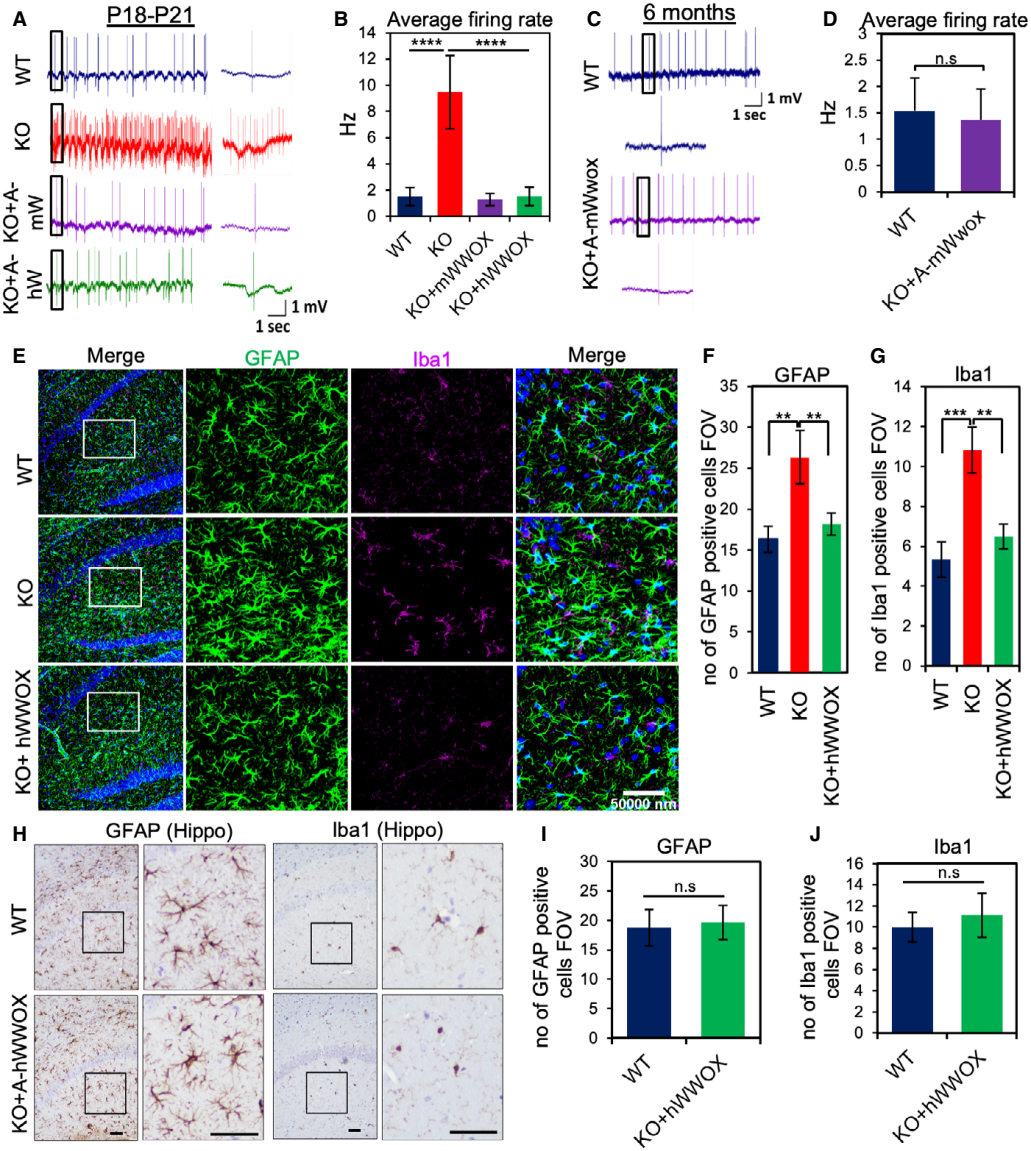
Neuronal restoration of WWOX reduces epileptic activity and astrogliosis in KO ARepresentative traces of cell‐attached recordings performed in WT (blue), KO (red), and KO injected with either with AAV9‐hSynI‐mWwox (KO+A‐mW shown in purple) or AAV9‐hSynI‐hWWOX (KO+A‐hW shown in green) pups at P18‐21 days. Traces represent spontaneous neocortical activity (action potentials shown). The panels represent 12‐s recording with an inset presenting a zoom‐in of a 0.5‐s interval.BThe graph shows the recordings (P18‐21) averages over 30 neurons from WT (*n* = 3), 30 neurons from KO+A‐mWwox (*n* = 3), 30 neurons from KO+A‐hWWOX (*n* = 3), and 45 neurons from KO (*n* = 3).CRepresentative traces of cell‐attached recordings performed in a WT (blue) and a KO+A‐mWwox (purple) adult mice (6 months old).DThe graph shows the averages over 60 neurons from WT adult mice (*n* = 3) and 60 neurons from KO+A‐mWwox adult mice (*n* = 3). There was no significant change observed between the average firing rate of the WT and the KO+A‐mWwox adult mice.EImmunofluorescence staining with anti‐GFAP (shown in green) and anti‐Iba1 (shown in magenta) antibodies showing the expression of astrocytes and microglia at P19 from WT, KO, and KO+A‐hWWOX. The square white box shows the magnified area from CA3 regions of hippocampus from all the genotypes.F, GGraphs represent the quantification of GFAP (F) and Iba1 (G) positive cells from the images shown in E. The number of GFAP or Iba1 cells was counted from 3 identical sagittal sections from CA3 region per group (*n* = 3 per each genotype).HImmunocytochemical images showing astrocytes (anti‐GFAP) and microglia (anti‐Iba1) in CA3 region of hippocampus from WT (*n* = 3) and rescued (KO+AAV‐hWWOX) (*n* = 3) mice at 9 months. Magnified area is shown with square box.I, JGraphs represent the quantification of GFAP (I) and Iba1 (J) positive cells from the images shown in H. The number of GFAP or Iba1 cells were counted from 3 identical sagittal sections (paraffin‐embedded) from CA3 region per group (*n* = 3 per each genotype). Data information: Error bars represent ± SD. Scale bars E) and H) 50 µm. n.s non‐significant (**P < 0.01, ***P < 0.001, ****P < 0.0001, Student’s *t*‐test).

We and others have previously reported that *Wwox*‐null mutants display spontaneous recurrent seizures (Suzuki *et al*, 2009; Mallaret *et al*, 2014; Cheng *et al*, 2020; Repudi *et al*, 2021). As we did not observe any spontaneous seizures in rescued mice, we determined next the neuronal hyperexcitability or epileptic activity in brains of P18‐21 wild type (WT), *Wwox* null (KO) either injected with AAV9‐mWwox or AAV9‐hWWOX, *Wwox* null alone by performing cell‐attached electrophysiology recordings (Appendix Fig S5). As expected, the KO pups exhibited severe hyperactivity; representative traces with spontaneous firing of action potentials are shown in Fig 3A. A clear hyperexcitability can be noted from the representative traces (WT in blue, KO in red, KO+AAV9‐mWwox in purple and KO+AAV9‐hWWOX in green). The activity of the KO brains usually resulted in bursts of action potentials, and overall, there was a drastic increase in the firing rate. The average firing rate over 30 WT, 30 KO+AAV9‐mWwox, 30 KO+AAV9‐hWWOX, and 45 KO recorded neurons was about 6‐fold higher in KO pups compared to the WT pups. No significant difference in average firing rate was observed between the KO+AAV9‐mWwox or KO+AAV9‐hWWOX and the WT pups (Fig 3B).

Since KO mice died within less than 4 weeks, we could not perform *in vivo* recordings in adult KO mice. We therefore performed cell attached *in vivo* recordings only in adult WT and KO+AAV9‐mWwox mice (Fig 3C). Representative traces are shown in blue (WT) and purple (KO+AAV9‐mWwox) and the average firing rate over 60 WT and 60 KO+AAV9‐mWwox neurons are presented (Fig 3D). There were no significant differences in the firing rate of adult WT and KO+AAV9‐mWwox cortical neurons. These findings indicate that AAV9‐hSynI‐WWOX vectors could prevent epileptic seizures resulting from WWOX loss.

In addition to the reduced neuronal hyperexcitability in the null mice, we also observed reduced activation of astrocytes and microglia in rescued KO mice at P19 (Fig 3E–G) and in older mice (9 months) (Fig 3H–J) hence indicating that neuronal WWOX restoration could reverse not only the neuronal hyperexcitability but also the associated neuroinflammatory phenotype.

### Neuronal restoration of WWOX enhances myelination in *Wwox*‐null mice likely by promoting OPC differentiation

**Figure 4:**
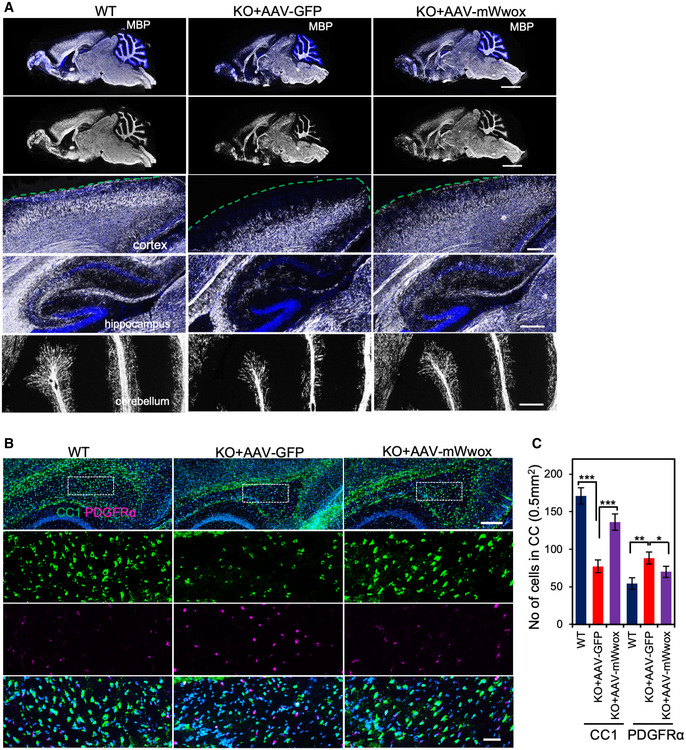
WWOX restoration in neurons improves myelination by promoting OPCs differentiation in *Wwox* null Images of whole‐brain sagittal sections that were immunolabeled with anti‐MBP at P17 from indicated mice (*n* = 3 for each group). MBP staining (shown in gray) in cortex (green dotted line represents the border of the cortex), hippocampus, and cerebellum is presented (magnified from the top panel).Sagittal section of the brain (P17) tissues immunostained for CC1 (green) and PDGFRα (magenta). Images showing increased number of matured oligodendrocytes in corpus callosum of the *Wwox* null after treatment with AAV‐mWwox compared to *Wwox* null injected with AAV‐GFP virus.Graph represents the quantification of CC1 and PDGFRα‐positive cells in corpus callosum (area 0.5 mm^2^) of WT (*n* = 3), KO (*n* = 3), and KO+A‐mWwox (*n* = 3) counted from three similar brain sagittal sections per mouse in each genotype. Data information: Error bars represent ± SD. (*P < 0.05, **P < 0.01, ***P < 0.001, Student’s *t*‐test). Scale bars A) 2 mm (top panel), 250 µm (middle and lower panels), B) 250 µm (top panel), 50 µm (middle and lower panels).

Previous observations linked WWOX loss with hypomyelination (Cheng *et al*, 2020; Repudi *et al*, 2021). In fact, it was shown that neuronal WWOX ablation results in a non‐cell autonomous function impairing differentiation of oligodendrocyte progenitors (OPCs) (Repudi *et al*, 2021). Hence, we next tested whether neuronal restoration of WWOX, using AAV, could rescue the hypomyelination phenotype in *Wwox*‐null mice. Immunofluorescence analysis of P17 sagittal brain tissues with anti‐MBP antibody revealed improved myelination in all parts (cortex, hippocampus, and cerebellum) of the rescued AAV9‐hSynI‐mWwox–treated mice brain compared to *Wwox*‐null mice injected with control virus (Fig 4A). Same results were obtained with AAV9‐hSynI‐hWWOX (Appendix Fig S6). In addition, we tested whether this improved myelination is associated with increased differentiation of OPCs to matured oligodendrocytes by a non‐cell autonomous function of neuronal WWOX. As expected, AAV9‐mediated WWOX expression in neurons increased the differentiation of OPCs to matured oligodendrocytes as assessed by immunostaining with CC1 (marker for matured oligodendrocytes) and anti‐PDGFRα (marker for OPCs) (Fig 4B). Quantification of CC1 and OPCs in the corpus callosum showed significantly increased number of matured oligodendrocytes in rescued mice compared to the KO mice injected with control virus at P17 (Fig 4C).

**Figure 5:**
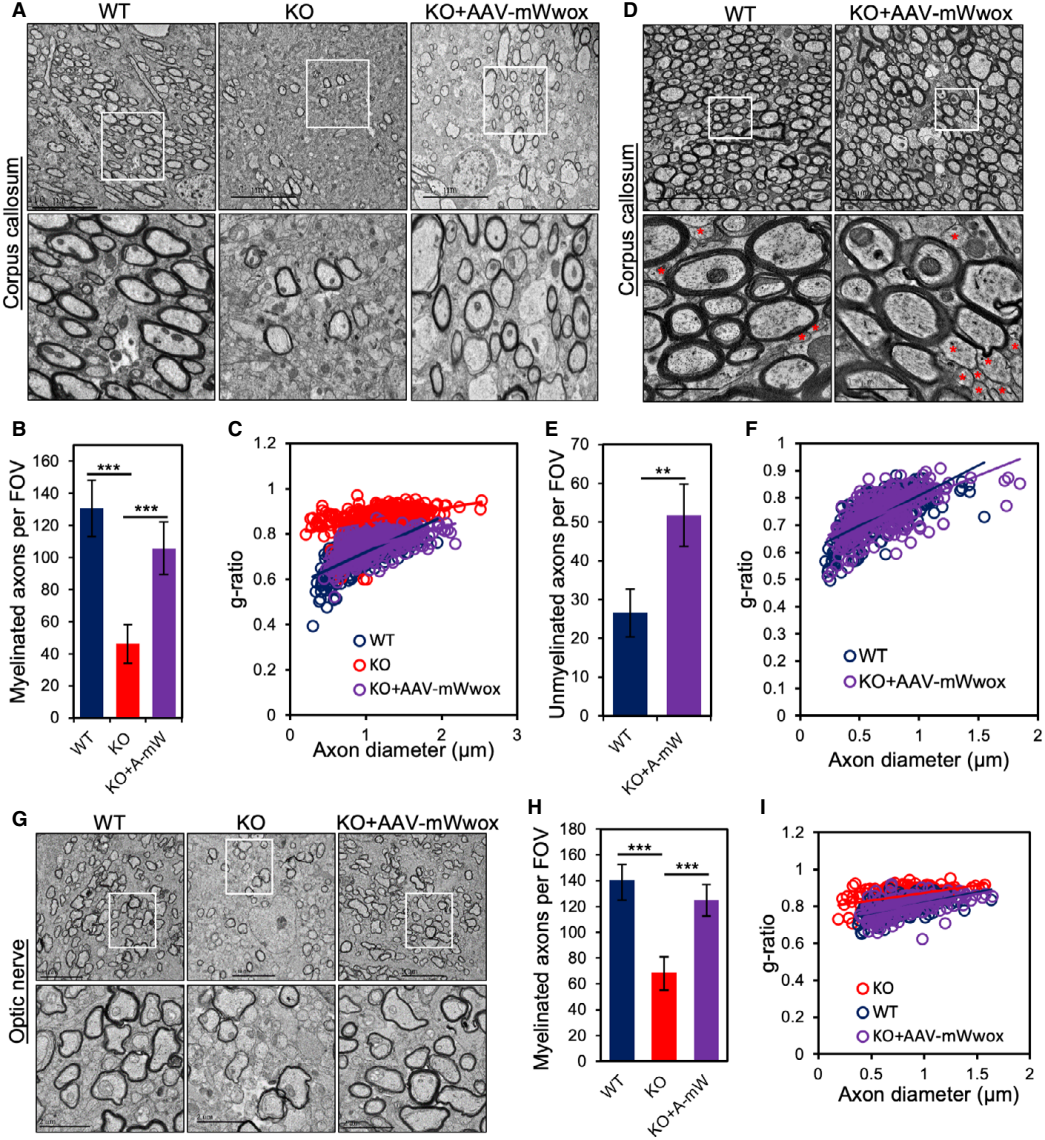
Electron microscopy analysis showing improved myelination in corpus callosum upon WWOX restoration Electron micrograph (EM) images from mid‐sagittal section of corpus callosum showing increased number of myelinated axons in AAV9‐hSynI‐mWwox‐injected mice compared to *Wwox*‐null mice (KO) at P17.Graph showing average number of myelinated axons in corpus callosum from WT (*n* = 3), KO (*n* = 3), or KO mice injected with AAV9‐hSynI‐mWwox (*n* = 3) per field of view (FOV).g‐ratio analysis showing reduced myelin thickness of axons in corpus callosum KO (*n* = 3) compared to the KO+AAV‐Wwox (*n* = 3). Axon diameter and myelin thickness were calculated from the electron micrograph images of corpus callosum (total no of axons *n* = 350 per each group).EM images from mid‐sagittal section of corpus callosum showing similar number of myelinated axons in rescued mice (AAV9‐hSynI‐mWwox, *n* = 3) compared to wild type (*n* = 3) at the age of 6 months. Unmyelinated axons are noted with the red star symbols in EM images.Graph represents the number of unmyelinated axons (corpus callosum) per FOV counted from representative EM images shown in D.Graph represents the measured g‐ratio of myelinated axons (*n* = 300 per each group) in WT and rescued mice at 6 months (*P* value 0.011334).EM images showing increased number of myelinated axons in optic nerve in AAV9‐hSynI‐mWwox‐injected mice compared to KO mice at P17.Graph showing the average number of myelinated axons (counted per FOV) in optic nerve from WT (*n* = 3), KO (*n* = 3), and KO+AAV‐mWwox(*n* = 3).g‐ratios of myelinated axons from optic nerves showing improved myelin thickness (less g‐ratio) after WWOX restoration (KO+AAV‐mWwox) compared to KO alone (*n* = 3 per genotype, total no of axons *n* = 200 per each group). Data information: Error bars represent ± SD. (**P < 0.01, ***P < 0.001, Student’s *t*‐test). Scale bars A) 10 µm (top panel), D) 5 µm (inset 1 µm), G) 5 µm (inset 2 µm).

To further validate the finding of improved myelination after neuronal restoration of WWOX, we performed electron microscopy (EM) analysis for corpus callosum at P17 and in adult mice. Remarkably, neuronal restoration of WWOX using AAV9‐hSynI‐mWwox increased the number of myelinated axons compared to KO at P17 (Fig 5A and B) in the corpus callosum. Furthermore, calculated g‐ratios indicated increased myelin thickness upon neuronal WWOX restoration compared to control KO mice (Fig 5C). In addition, EM images of the corpus callosum of adult (6 months) rescued mice showed improved myelination (Fig 5D–F). Of note, when comparing myelin thickness of KO+AAV‐mWwox and WT corpus callosum at P17 and 6 months, we observed some minor differences in the g‐ratio and the number of unmyelinated axons (Fig 5C, E and F). Additionally, we also performed EM analysis of optic nerves at P17 of AAV9‐WWOX‐treated mice which revealed increased number of myelinated axons as well as myelin thickness (Fig 5G–I). Cumulatively, neuronal restoration of WWOX positively regulates myelination likely by promoting OPC differentiation in a non‐cell autonomous manner.

### Behavioral and motor functions of *Wwox*‐null mice upon neuronal WWOX restoration

**Figure 6:**
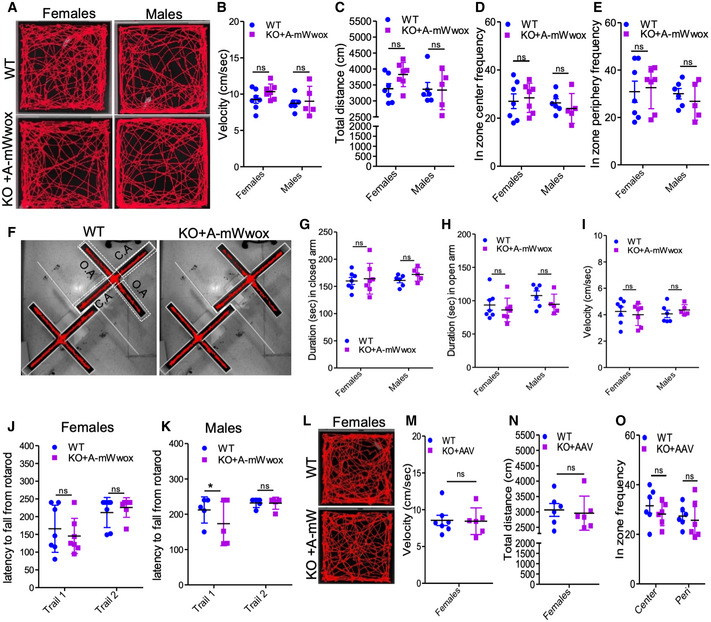
WWOX restoration in neurons reverses the abnormal behavioral phenotypes of *Wwox*‐null mice ARepresentative images of open‐field test showing tracking pattern (open arena) of WT (females, *n* = 7, males *n* = 6) and AAV‐mWwox‐injected KO mice (females *n* = 7, males *n* = 5) at 8–10 weeks.B–EDot plot graphs represent the velocity (B), total distance traveled (C), the frequency to enter into the center (D), and periphery (E) in an open arena from the open field tracking as shown in A.FMice tracking images of elevated plus maze test from WT and KO+AAV‐mWwox mice (age 8–10 weeks). O.A. (open arm) C.A. (closed arm) are shown with dotted white boxes on the left mice tracking image.G–IDot plot graphs represent the duration (sec) in closed arm (G), open arm (H), and movement velocity (I) from WT (females, *n* = 7, males *n* = 6) and KO+AAV‐mWwox (females *n* = 7, males *n* = 5).J, KGraphs represent the latency (sec) of the mice (WT and rescued mice at 8–10 weeks) to fall from the rotarod. WT (females, *n* = 7, males *n* = 6) and KO+AAV‐Wwox (females *n* = 7, males *n* = 5).LRepresentative images of open field test showing tracking pattern (open arena) of WT (females, *n* = 6) and KO with AAV injected (females mWwox *n* = 3, hWWOX *n* = 3) at 8–9 months.M–ODot plot graphs represent the represent the velocity (M), total distance traveled (N), the frequency to enter into the center and periphery (O) in an open arena from the open field tracking of mice shown in L. Data information: Error bars represent ± SD. n s (no significance) (*P < 0.05). Statistical analysis was done using two‐way ANOVA with Bonferroni post hoc comparisons.

We next explored the behavioral changes in *Wwox‐*null mice after restoration of WWOX in neurons. Unfortunately, we could not assess behavior of *Wwox*‐null mice due to their poor conditions and premature death. We hence performed open‐field, elevated plus maze (EPM), and rotarod tests to examine anxiety and motor coordination in WT and rescued mice (Fig 6). Remarkably, at 8–10 weeks, we observed similar tracking patterns in open field in the rescued mice (males and females) to those seen in WT mice (Fig 6A). In addition, the velocity, total distance traveled in the open field tracks and the frequencies to enter into the center or to the periphery of open arena were very similar to that of the WT mice (Fig 6B–E). Moreover, rescued female and male mice exhibited near normal behavior in the EPM test (Fig 6F–I). Rotarod test was performed to check the motor coordination in rescued mice, and our results revealed that rescued mice had similar motor coordination to WT mice at different trails, an indicative of their learning ability (Fig 6J and K). An open‐field experiment was also performed on some aged mice (8–9 months) and revealed no difference between WT and rescued mice (Fig 6L–O). Altogether, these results suggest that neuronal WWOX re‐expression in *Wwox*‐null mice restores normal activity and behavior.

## Discussion

Monogenic neurological disorders have been estimated to account for up to 40% of the workload in hospital pediatric practice. Mostly, the etiopathologies of these disorders are complex and are often untreatable. WOREE syndrome is one of these complex neurological and developmental disorders having no cure or proper treatment so far. Based on our recent findings (Repudi *et al*, 2021), we aimed here to restore WWOX in neurons and assess the therapeutic potential of this restoration. In this study, we utilized an AAV9 vector for targeted gene delivery of WWOX to mature neurons to treat the complex neuropathy in the *Wwox*‐null mouse model. We injected *mWwox* or *hWWOX cDNA* under the neuronal promotor *Synapsin‐I* into the brains of newborn *Wwox*‐null mice and showed that this treatment was able to reverse the phenotypes of WWOX deficiency.

The role of WWOX in regulating CNS homeostasis is emerging as a key function of the *WWOX* gene. Deficiency of WWOX has been linked to a number of neurological disorders (Aldaz & Hussain, 2020; Banne *et al*, 2021). Of particular interest is WOREE syndrome, a devastating complex neurological disease‐causing premature death with a median survival of 1–4 years (Banne *et al*, 2021). WOREE children are refractory to the current antiepileptic drugs (AEDs), hence challenging the medical and scientific communities to develop new therapeutic strategies. We believe that delivering AAV9‐WWOX into the brain of WOREE syndrome patients could be a novel gene therapy approach that would help these patients. Recent success in gene therapy clinical trials of the treatment of spinal muscular atrophy (SMA) using the AAV9 vector (Pattali *et al*, 2019) is encouraging and has promoted our further development of this platform in this proof‐of‐concept study.

The effects of delivering AAV9‐SynI‐WWOX (murine or human sequence) into the brains of *Wwox*‐null mice were remarkable. Firstly, WWOX neuronal delivery restored normal growth, activity and fertility, and survival of mice with no occurrence of spontaneous seizures and ataxia. In addition, we showed that neuronal restoration of WWOX reduced hyperexcitability in cell‐attached recordings. Secondly, neuronal WWOX restoration improved myelination of all regions of the brain further confirming the previous observations of WWOX neuronal non‐cell autonomous function on OPC maturation (Repudi *et al*, 2021). Of note, there are still some differences between rescued and WT mice which could be attributed to an oligodendrocyte‐specific WWOX function in regulating the myelination process. Thirdly, WWOX restoration improved the overall behavior of the rescued mice. These findings might suggest that WWOX’s proposed role in regulating autism (Mignot *et al*, 2015; Peter *et al*, 2019; Piard *et al*, 2019; Aldaz & Hussain, 2020; Banne *et al*, 2021) and perhaps other behavior‐associated disorders is driven by proper neuronal function of WWOX. It should be noted that our analyses did not reveal any difference between mWwox and hWWOX vectors.

Another intriguing consequence of neuronal WWOX delivery is the reversibility of hypoglycemia associated with WWOX deficiency in *Wwox*‐null mice (Abu‐Remaileh & Aqeilan, 2014; Abu‐Remaileh *et al*, 2014). Our results are consistent with a central role of WWOX in the CNS regulation of metabolism of glucose and likely other metabolic functions (Lee *et al*, 2008; Iatan *et al*, 2014; Abu‐Remaileh & Aqeilan, 2015; Abu‐Remaileh *et al*, 2018). Interestingly, targeted deletion of *Wwox* in skeletal muscle resulted in impaired glucose homeostasis (Abu‐Remaileh *et al*, 2019), and this effect was linked to cell‐autonomous functions of WWOX.

One more peculiar observation is that the rescued mice were also fertile and able to breed. Given that *Wwox*‐null mice were previously shown to display impaired steroidogenesis (Aqeilan *et al*, 2009; Ludes‐Meyers *et al*, 2009; Saluda‐Gorgul *et al*, 2011) and no peripheral expression of WWOX in the rescued mice was observed (Appendix Fig S2), our findings imply that WWOX’s function in the CNS is superimposing its tissue‐level function. Altogether, these findings suggest that WWOX could have pleotropic function both at organ and organism levels.

WWOX is ubiquitously expressed in all brain regions (Chen *et al*, 2004; Chiang *et al*, 2013; Aldaz & Hussain, 2020). Our current observations do not imply that WWOX expression in other brain cell types, such as astrocytes and oligodendrocyte, are dispensable. Evidence linking WWOX function with oligodendrocyte pathology is starting to emerge (International Multiple Sclerosis Genetics C, 2019; International Multiple Sclerosis Genetics C *et al*, 2013; Jakel *et al*, 2019; Matsushita *et al*, 2015; Ziliotto *et al*, 2019); however, less is known about the cell‐autonomous functions of WWOX in oligodendrocytes. The fact that WWOX expression in neurons regulates oligodendrocyte maturation and antagonizes astrogliosis (Hussain *et al*, 2019) suggests a complex function of WWOX in CNS physiology and pathophysiology that warrants further in‐depth analysis.

The *WWOX* gene was initially cloned as a putative tumor suppressor (Bednarek *et al*, 2000; Ried *et al*, 2000). Indeed a plethora of research work in various animal models (reviewed in (Tanna & Aqeilan, 2018)) and observations in human cancer patients (Kurek *et al*, 2010; Aldaz *et al*, 2014; Gardenswartz & Aqeilan, 2014; Baryla *et al*, 2015; Abu‐Remaileh *et al*, 2018; Abdeen & Aqeilan, 2019; Khawaled *et al*, 2019, 2020) proposed WWOX as a tumor suppressor. Given that our restoration of WWOX is limited to brain, we assumed other tissues lacking WWOX expression would be more susceptible to tumor development. Of note, we did not detect gross tumor formation in the limited number of adult *Wwox*‐null mice treated with AAV9‐hSynI‐WWOX that we examined (age 8–11 months). This is not surprising and consistent with data showing that *Wwox* somatic deletion in several tissues required other hits to promote tumor formation in animal models (Ferguson *et al*, 2012; Abdeen, Salah, *et al*, 2013; Del Mare *et al*, 2016; Abu‐Remaileh *et al*, 2018). Nevertheless, detailed cellular and molecular analyses shall be required in the future to further investigate any abnormal changes associated with WWOX deficiency in AAV‐treated mice.

The limited life span and poor conditions of *Wwox*‐null mice prompted us to treat these mice very early on in their life (P0). Nevertheless, attempts to treat post‐natal *Wwox*‐null mice by different route of AAV administration should and will be explored in the future.

In summary, our current findings indicate that WWOX restoration in mature neurons of neonatal mice using an AAV vector could reverse the phenotypes associated with WWOX deficiency. We envisage that this proof‐of‐concept will lay down the groundwork for a possible gene therapy clinical trial on children suffering from the devastating and often refractory WOREE and SCAR12 syndromes.

## Materials and Methods

### Plasmid vectors

Murine *Wwox* or human *WWOX* cDNA was cloned under the promoter of human *Synapsin I* in pAAV, and this vector was packaged into AAV9 serotype (Vector Biolabs, Philadelphia, USA). Custom‐made AAV9‐hSynI‐mWwox‐IRES‐EGFP, AAV9‐hSynI‐hWWOX, and AAV9‐hSynI‐EGFP viral particles were obtained either from Vector Biolabs or from the Vector Core Facility at Hebrew University of Jerusalem. Viral titer was measured by qRT–PCR using bGH primers.

### Mice

Generation of *Wwox*‐null ^(−/−)^ mice (KO) was previously reported (Aqeilan *et al*, 2009), and these mice were maintained in an FVB background. Heterozygote ^(+/−)^ mice were used for breeding to get the *Wwox*‐null mice. Wild‐type and the rescued mice (KO injected either with AAV‐mWwox or AAV‐hWWOX) were separated at weaning based on their sex then housed in individual ventilated cages (IVC). Animals were maintained in a SPF (specific pathogen‐free) unit in a 12 h‐light/dark cycle with *ad libitum* access to the food and water. All animal‐related experiments were performed in accordance and with prior approval of the Hebrew University‐Institutional Animal Care Use Committee (HU‐IACUC).

### DRG culture

DRG (dorsal root ganglion) neurons were cultured following a previously published protocol (Repudi *et al*, 2021). Briefly, DRG neurons were isolated from mouse embryos at E13.5. Embryos were genotyped and the DRGs were collected in cold L‐15 medium. Tissues were dissociated in 0.25% trypsin, triturated, centrifuged, and re‐suspended in NB medium (Neurobasal, B27 supplement, 0.5 mM l‐glutamine, and penicillin–streptomycin). Pre‐cleaned 13‐mm diameter glass coverslips were placed in 4‐well dishes and coated with Matrigel (1 h at RT) then poly‐D‐lysine (30 min at RT) prior to dissection. Cells were plated at a density of 40,000 cells/13‐mm coverslips in NB medium and maintained in a humidified incubator at 37°C and 5% CO_2_. Cultures were treated with fluorodeoxyuridine at DIV2, 4, and 6 to eliminate non‐neuronal cells. Fifty percent of cell media was replaced every third day. At DIV6, *Wwox*‐null DRG neurons were infected with either AAV‐GFP or AAV9‐mWwox and cultured for 3 days, and then, expression of GFP and WWOX were validated by immunofluorescence.

### Intracerebroventricular (ICV) injection of AAV particles into P0 *Wwox*‐null mice

Free‐hand intracranial injections of either AAV9‐hSynI‐mWwox‐IRES‐EGFP, AAV9‐hSynI‐hWWOX, or AAV9‐hSynI‐EGFP *(*AAV9‐GFP*)* into the *Wwox‐*null mice were done following a published protocol (Kim *et al*, 2014). Briefly, when neonates were born, they were PCR genotyped to identify *Wwox*‐null mice. *Wwox*‐null neonates were anesthetized by placing on a dry, flat, cold surface. The anesthetized pup head was gently wiped with a cotton swab soaked in 70% ethanol. Trypan blue 0.1% was added to the virus to enable visualization of the dispensed liquid. An injection site was located at 2/5 of the distance from the lambda suture to each eye. Holding the syringe (preloaded with virus) perpendicular to the surface of the skull, the needle was inserted to a depth of approximately 3 mm. Approximately 1 µl (2 × 10^10^ GC/hemisphere) virus was dispensed using a NanoFil syringe with a 33G beveled needle (World Precision Instruments). The other hemisphere was injected in the same way. Injected pups were placed on the warming pad until they were awake and then transferred to the mother’s cage. Each injected mouse was carefully monitored for growth, mobility, seizures, ataxia, and general condition to assess phenotypes.

### Weight and blood glucose levels

Mice were weighed regularly as indicated in the Figures. To monitor the blood glucose, the tip of the mouse tail was ruptured with scissors and a tiny drop of blood collected for measurement (mg/dl) using an Accu‐Check glucometer (Roche Diagnostics, Mannheim, Germany).

### Immunofluorescence

Mice from different genotypes and treatment groups were euthanized by CO_2_ and transcardially perfused with 2% PFA/PBS. Dissected brains were postfixed on ice for 30 min and then incubated in 30% sucrose at 4°C overnight. They were then embedded in OCT and sectioned (12–14 µm) using a cryostat. Sagittal sections were washed with PBS and blocked with 5% goat serum containing 0.5% Triton X‐100 and then incubated for 1 h at room temperature followed by incubation with primary antibodies overnight at 4°C. Then, sections were washed with PBS and incubated with corresponding secondary antibodies tagged with Alexa fluorophore for 1 h at room temperature followed by washing with PBS and mounting with mounting medium. List of all the primary and secondary antibodies and the dilutions used in this study are provided in a table (Appendix Table S1).

### Immunohistochemistry

Tissues collected from different mice at different ages were fixed in 4% formalin. Paraffin‐embedded tissue sections were deparaffinized and rehydrated. Antigen retrieval was performed in 25 mM sodium citrate buffer pH 6.0 (for WWOX, GFAP, Iba1) using pressurized chamber for 2.5 min. Endogenous peroxidase was blocked with 3% H_2_O_2_ for 15 min. The sections were then incubated with blocking solution (CAS Block) for 30 min to reduce non‐specific binding followed by incubation with the primary antibody. Slides were subsequently incubated with horseradish peroxidase‐conjugated anti‐rabbit or anti‐mouse immunoglobulin antibody for 30 min. The enzymatic reaction was detected in a freshly prepared 3,3 diamminobenzidine using DAB peroxidase kit (Vector laboratories) for several min at room temperature. The sections were then counterstained with hematoxylin.

### Surgical procedures for electrophysiology

Mice were anesthetized using ketamine/medetomidine (i.p; 100 and 83 mg/kg, respectively). The effectiveness of anesthesia was confirmed by the absence of toe‐pinch reflexes. Supplemental doses were administered every ˜1 h with a quarter of the initial dosage to maintain anesthesia during the electrophysiology procedures. During all surgeries and experiments, body temperature was maintained using a heating pad (37°C). The skin was removed to expose the skull. A custom‐made metal pin was affixed to the skull using dental cement and connected to a custom stage. A small hole (3 mm diameter craniotomy) was made in the skull using a biopsy punch (Miltex, PA).

### Cell‐attached recordings

Cell‐attached recordings were obtained with blind patch‐clamp recording. Electrodes (˜7 MOhm) were pulled from filamented, thin‐walled, borosilicate glass (outer diameter, 1.5 mm; inner diameter, 0.86 mm; Hilgenberg GmbH, Malsfeld, Germany) on a vertical two‐stage puller (PC‐12, Narishige, EastMeadow, NY). The electrodes were filled with internal solution containing the following: 140 mM K‐gluconate, 10 mM KCl, 10 mM HEPES, 10 mM Na_2_‐phosphocreatine, and 0.5 mM EGTA, adjusted to pH 7.25 with KOH. The electrode was inserted at a 45 degrees angle and reached a depth of 300 µm. The electrode positioning was targeted on the brain surface, positioned at 1.6–2 mm posterior to the bregma and 4 mm lateral to the midline. While positioning the electrode, an increase of the pipette resistance to 10–200 MOhm resulted in most cases in the appearance of action potentials (spikes). The detection of a single spike was the criteria to start the recording. All recordings were acquired with an intracellular amplifier in current clamp mode (Multiclamp 700B, Molecular Devices), acquired at 10 kHz (CED Micro 1401‐3, Cambridge Electronic Design Limited) and filtered with a high pass filter. For calculation of the average firing rate, the firing rate over a 4‐min recording period was calculated for each of the recorded cells.

### Electron microscopy

Mice were anesthetized and perfused with a fixative containing 2% paraformaldehyde and 2.5% glutaraldehyde (EM grade) in 0.1 M sodium cacodylate buffer, pH 7.3. Brains were isolated and incubated in the same fixative for 2 h at room temperature and then stored in 4°C until they were processed. Collected tissues (corpus callosum, optic nerve) were washed four times with sodium cacodylate and postfixed for 1 h with 1% osmium tetroxide, 1.5% potassium ferricyanide in sodium cacodylate, and washed four times with the same buffer. Then, tissue samples were dehydrated with graded series of ethanol solutions (30, 50, 70, 80, 90, 95%) for 10 min each and then 100% ethanol three times for 20 min each, followed by two changes of propylene oxide. Tissue samples were then infiltrated with series of epoxy resin (25, 50, 75, 100%) for 24 h each and polymerized in the oven at 60°C for 48 h. The blocks were sectioned by an ultramicrotome (Ultracut E, Riechert‐Jung), and sections of 80 nm were obtained and stained with uranyl acetate and lead citrate. Sections were observed using a Jeol JEM 1400 Plus transmission electron microscope, and pictures were taken using a Gatan Orius CCD camera. EM micrographs were analyzed using computer‐assisted ImageJ analysis software. To calculate g‐ratio, myelinated axons (˜300, 100 axons per mouse, *n* = 3 per genotype) from EM images from corpus callosum were analyzed by dividing inner axonal diameter over the total axonal diameter.

### Open‐field test

The open‐field test was performed following the previously published protocol (Wolf *et al*, 2018). Briefly, mice were placed in the corner of a 50 × 50 × 33 cm arena and allowed to freely explore for 6 min. The center of the arena was defined as a 25 × 25 cm square in the middle of the arena. Velocity and time spent in the center and arena circumference were measured. Mice tested in the open field were recorded using a video camera connected to a computer having tracking software (Ethovision 12).

### Elevated plus maze test

The test apparatus consisted of two open arms (30 × 5 cm) bordered by a 1 cm high rim across from each other and perpendicular to two closed arms bordered by a rim of 16 cm, all elevated 75 cm from the floor. Mice were put into the maze and were allowed to explore it for 5 min. Duration of visits in both the open and closed arms was recorded (Wolf *et al*, 2018).

### Rotarod test

Each animal was placed on a rotating rod whose revolving speed increased from 5 rounds per min (rpm) to 40 rpm for 99 s. The test for each animal consisted of three trials separated by 20 min. The initial trial was considered as training for mice and the results from the last two trails were considered. Time to fall from device (latency) was recorded for each trial for each animal. If the animal did not fall from the device by 240 s from the beginning of the trial, the trial was terminated (Greenbaum *et al*, 2012).

### Image acquisition and analysis

Immunostained sections were imaged using a Nikon A1R+ confocal microscope or an Olympus FV1000 confocal laser scanning microscope or panoramic digital slide scanner. The acquired images were processed using the associated microscope software programs, namely CaseViewer, F‐10‐ASW viewer, and NIS elements, respectively. Images were analyzed using ImageJ software. Images were analyzed while blinded to the genotype and the processing included the global changes of brightness and contrast.

### Statistical analysis

All graphs and statistical analyses were performed using either Excel or GraphPad Prism 5. Results of the experiments were presented either as mean ± SEM or ± SD (mentioned in the figure legends). The two‐tailed unpaired Student’s *t*‐test or two‐way ANOVA with Bonferroni for post hoc comparisons was used to calculate the significance. Results were considered significant when the *P* < 0.05, otherwise they were represented as ns (no significance). Data analysis was performed while blinded to the genotype. The statistical test used, sample size, and *P* value are indicated in the figure legends.

## Author contributions

Conceptualization, SR and RIA; Methodology, SR, SA‐S, and IK; Investigation, SR, IK, and SA‐S; Writing—Review and Editing, SR SS, and RIA; Funding Acquisition, RIA; Resources, SR and SA‐S; Project Administration, RIA; Supervision, SS and RIA.

## Conflict of interest

The authors declare that they have no conflict of interest.

## For more information

- OMIM website of DEE28 at https://www.omim.org/entry/616211
- Human disease genes website at https://humandiseasegenes.nl/wwox/
- Visit the “WWOX Foundation” official website at https://www.wwox.org/

## Supporting information



Appendix

## Data Availability

This study includes no data deposited in external repositories.
