# Supplementary material for: Neonatal neuronal WWOX gene therapy rescues Wwox null phenotypes
Source: EMBO Mol Med. 2021 Nov 7;13(12):e14599. doi: 10.15252/emmm.202114599 (PMC8649866; doi:10.15252/emmm.202114599)
Supplement: Supplementary file 1 — Appendix [file EMMM-13-e14599-s001.pdf]

List of contents (Appendix)

Appendix Figure S1
Appendix Figure S2
Appendix Figure S3
Appendix Figure S4
Appendix Figure S5
Appendix Figure S6
Appendix Table S1

Appendix Fig S1

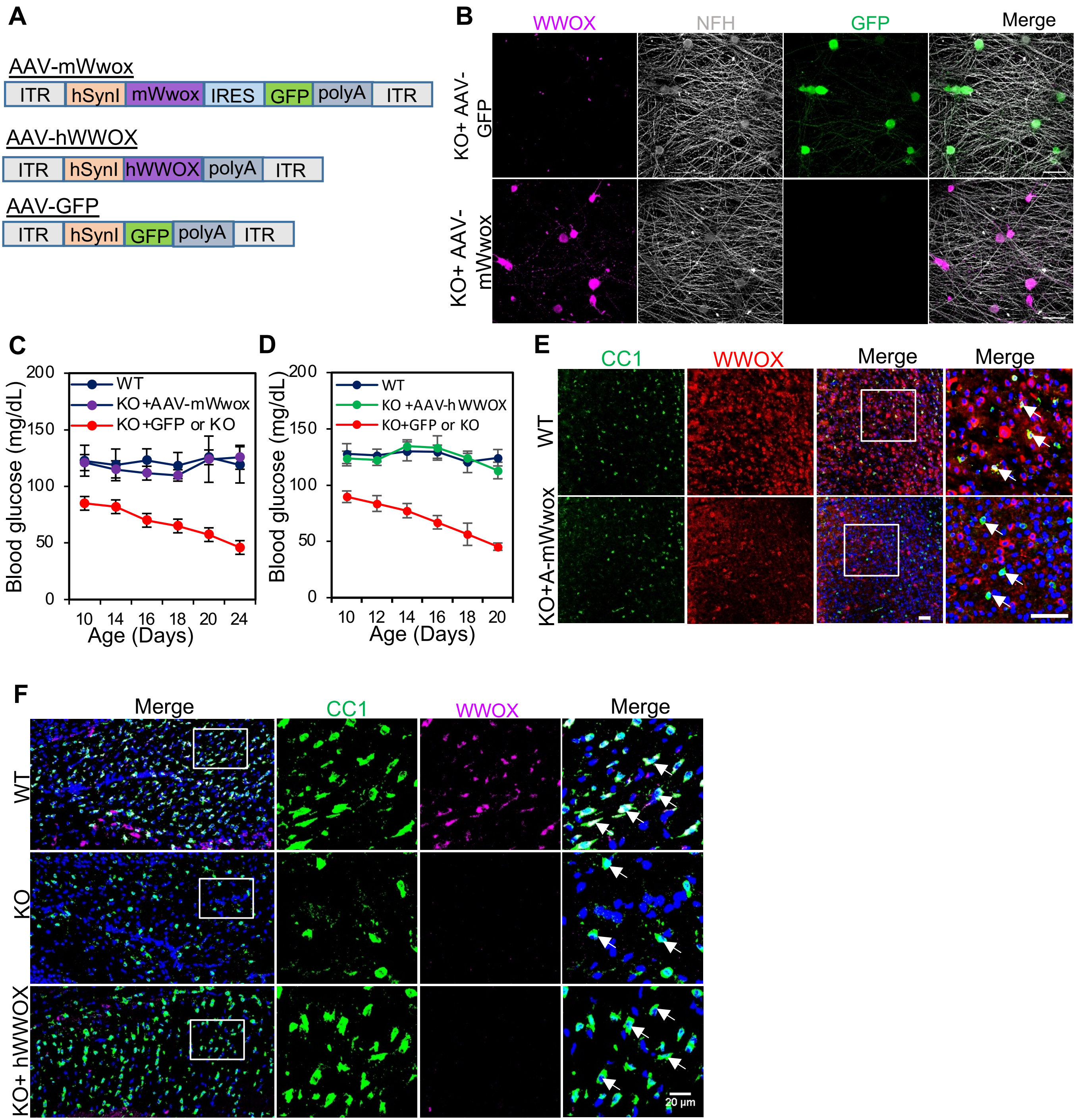

Appendix Fig S2

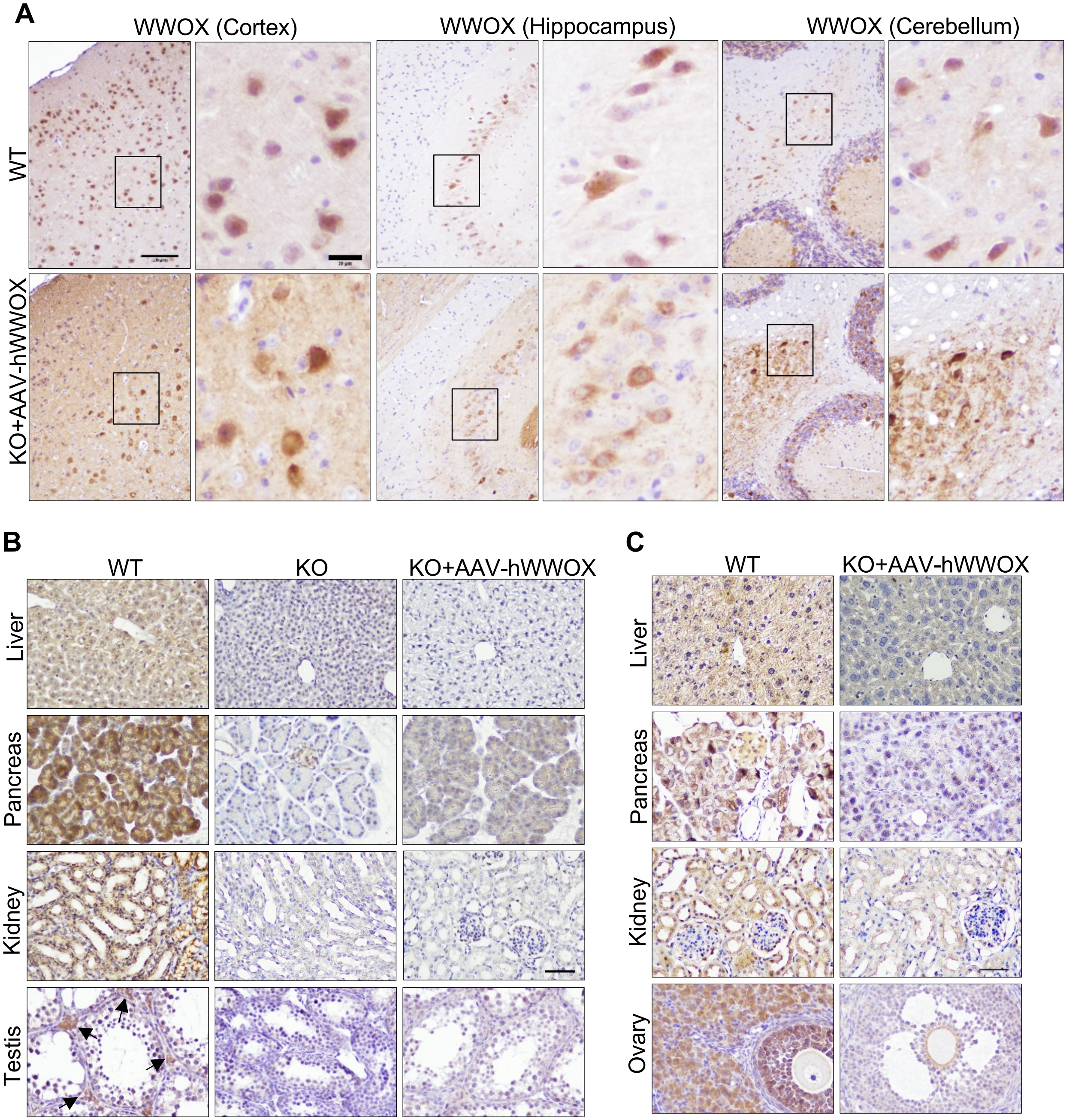

Appendix Fig S3

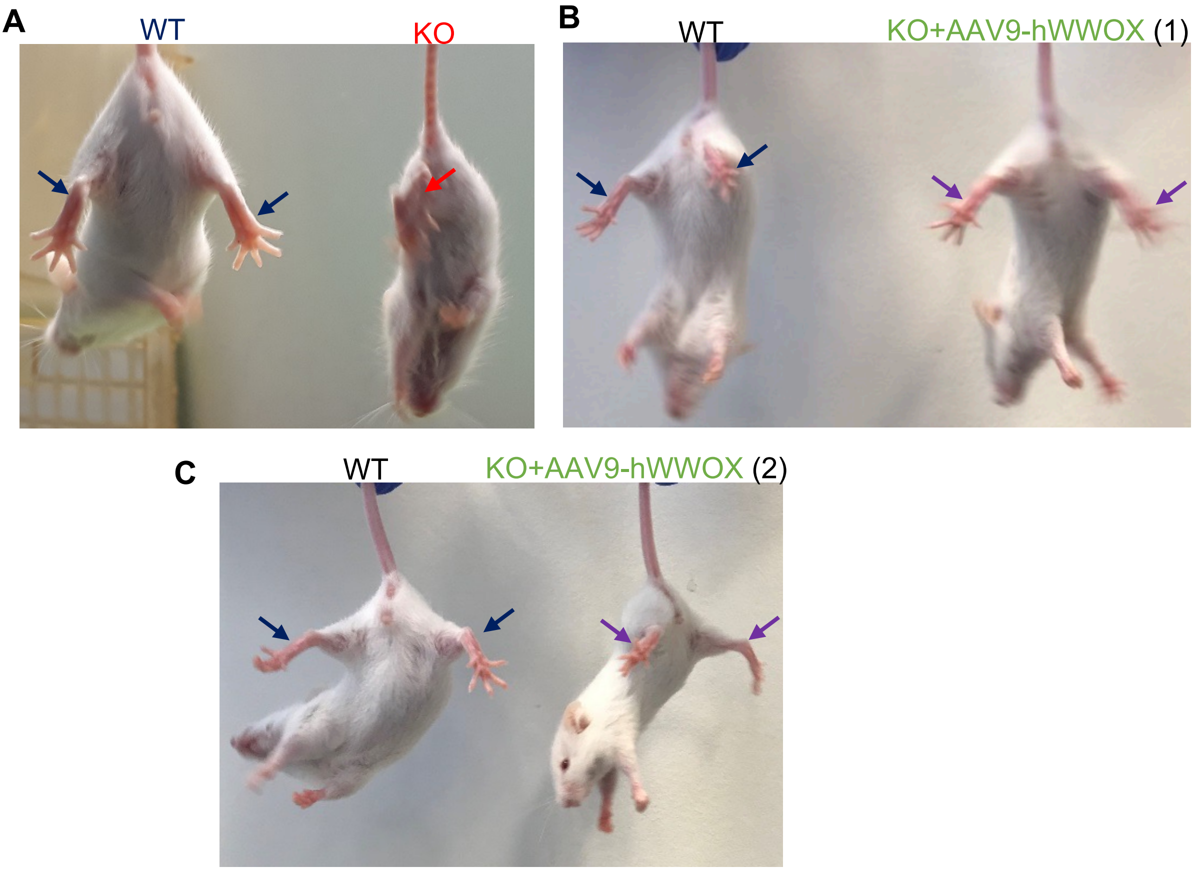

Appendix Fig S4

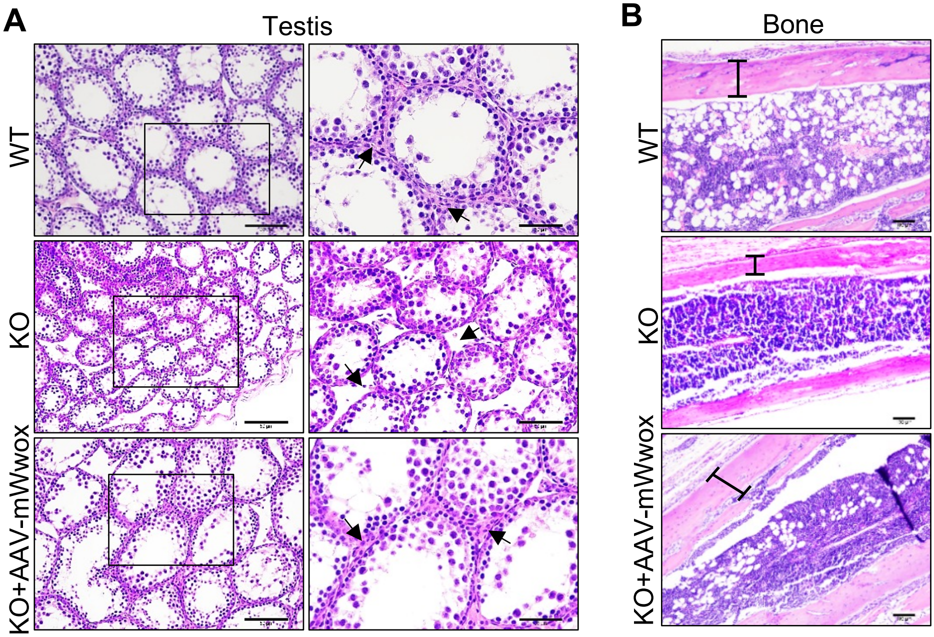

Appendix Fig S5

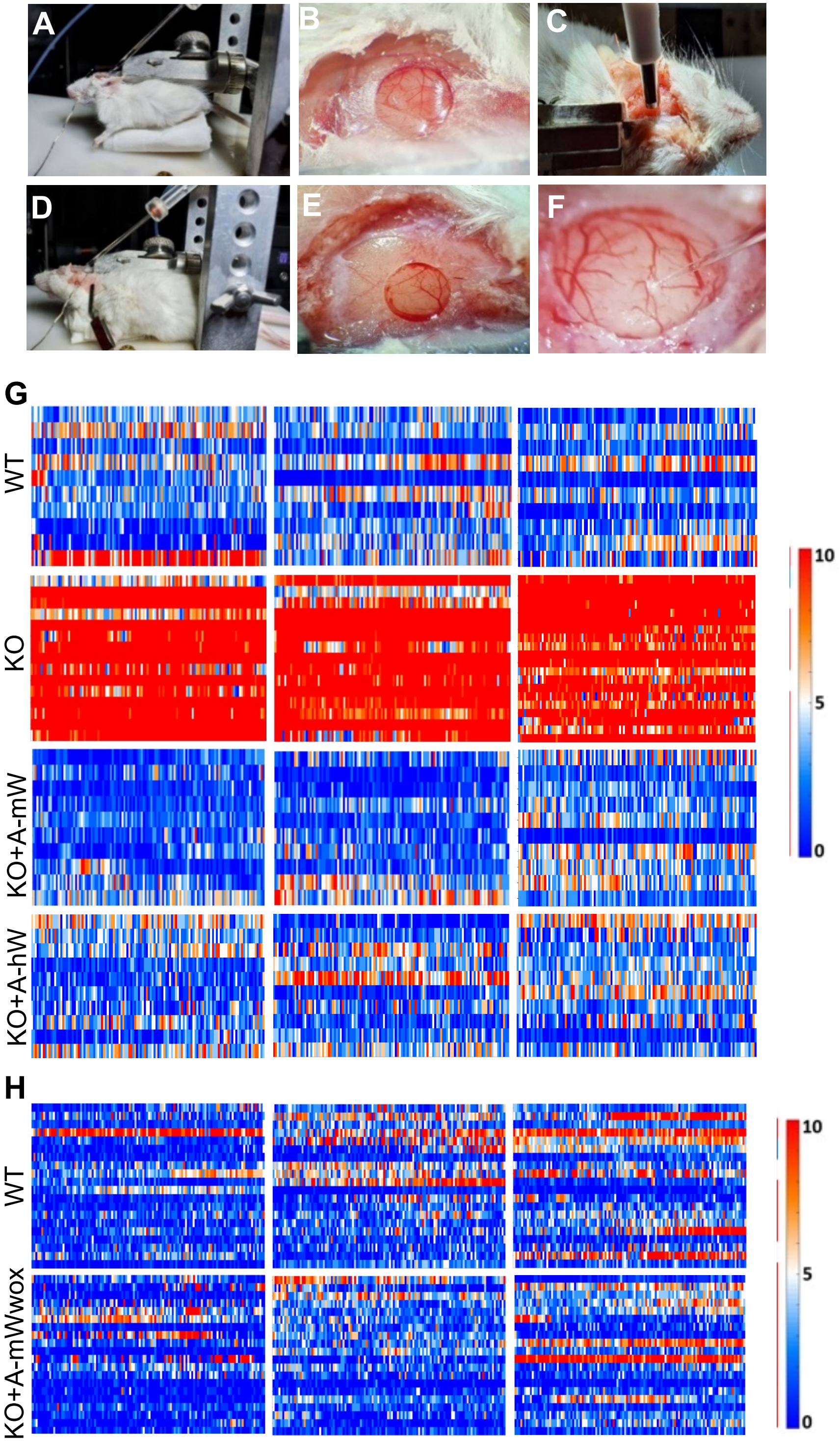

Appendix Fig S6

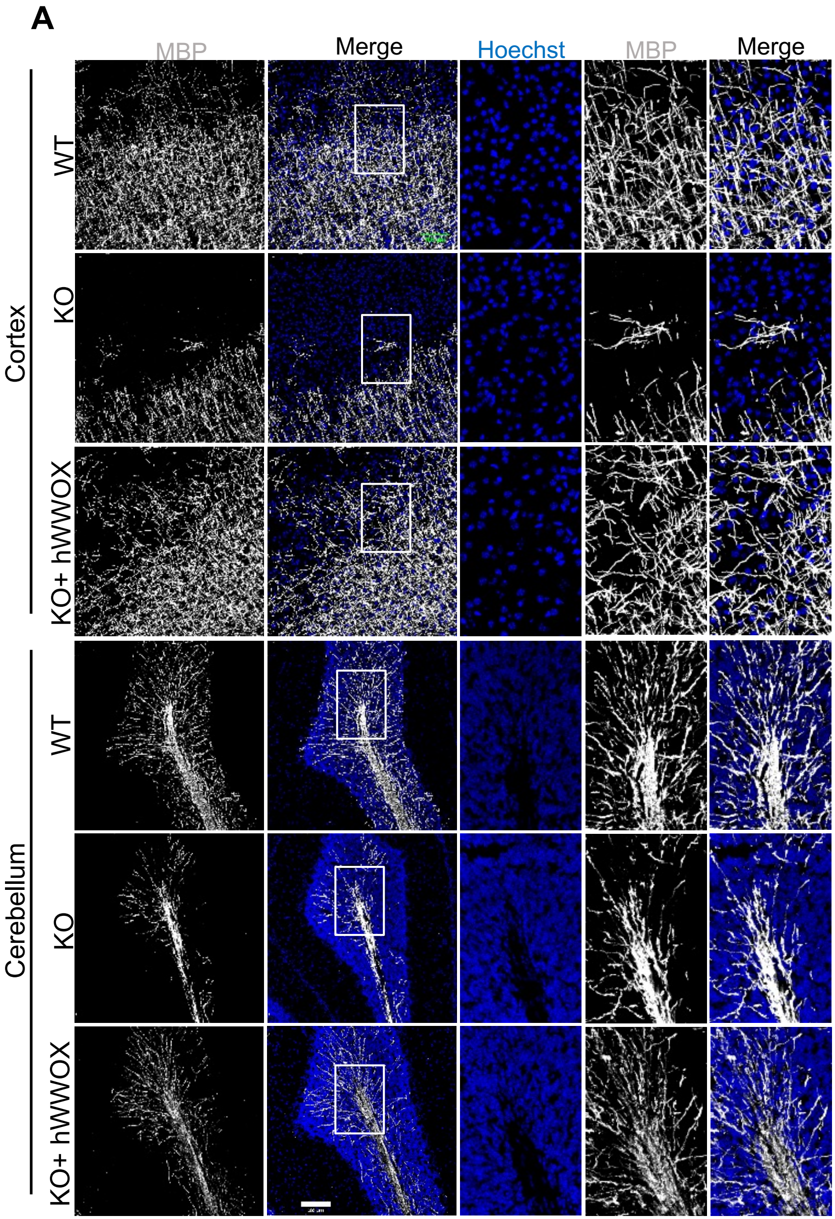

Appendix Table S1

List of antibodies and their dilutions used in the current study

Immunofluorescence (IF) **Primary Antibodies**	**Dilution of the antibody**	**Cat No**	**Source**
Mouse anti-CC1	1:50	OP80	Millipore
Rabbit anti-WWOX	1:5000	N.A	N.A
Rat anti-PDGFRα	1:500	558774	BD Pharmingen
Mouse anti-NeuN	1:500	MAB377	Millipore
Rabbit anti-MBP	1:100	Ab65988	Abcam
Rat anti-Neurofilament H	1:100	MAB5448	Millipore
Mouse anti-GFAP	1:500	MAB360	Millipore
Rabbit anti-Iba1	1:500	019-19741	Wako
**Secondary antibodies**			
Goat anti-Mouse IgG, alexa fluor 488	1:1000	A11029	Invitrogen
Goat anti-Mouse IgG, alexa fluor 647	1:1000	A21244	Invitrogen
Donkey anti-Rat IgG, conjugated with Cy5	1:500	712-175-150	Jackson Immuno Research
Immunohistochemistry (IHC)			
**Primary Antibodies**			
Mouse anti-GFAP	1:1000	MAB360	Millipore
Rabbit anti-Iba1	1:1000	019-19741	Wako
Rabbit anti-WWOX	1:10000	N.A	N.A
**Secondary antibodies**			
Mouse IgG conjugated with HRP	Direct	MP-7402	Vector laboratories
Rabbit IgG conjugated with HRP	Direct	MP-7401	Vector laboratories

Appendix Figure legends

Appendix Fig S1. Validating the expression of mWwox and hWWOX *ex vivo* and *in vivo*.

A. Schematic representation of AAV vector constructs containing murine *Wwox* or human *WWOX* or GFP under human Synapsin I promoter. The m*Wwox* gene sequence is followed by an IRES and *EGFP* gene sequence.

B. Cultured primary *Wwox* null dorsal root ganglion (DRG) neurons were infected with AAV-GFP or AAV-mWwox. Immnuofluorescence images showing the expression of GFP (auto fluorescence) and WWOX (with anti-WWOX antibody). Neurofilaments are stained with anti-NFH antibody (shown in gray).

C, **D**. Graphs represent the blood glucose levels of WT, KO, KO+ AAV-mWwox (C) or KO+ AAV-hWWOX (D) at indicated days (n=5 per each group).

E, **F**. Brain sections (P17) were immunolabelled with CC1 (oligodendrocytes) and anti-WWOX antibodies. Images showing WWOX expression in oligodendrocytes (shown with arrows in WT panel) of WT and not in *Wwox* null (shown with arrows) injected with AAV-mWwox (**E**) or AAV-hWWOX (**F**). Representative images are shown from the cortex (**E**) and corpus callosum (**F**).

Data information: Error bars represent ±SEM. Scale bars **B**) 10 µm and **E**) 30 µm**, F**) 20 µm.

Appendix Fig S2. Validating the expression of WWOX in peripheral tissues and brain at different ages.

A. Immunocytochemical images showing the expression of WWOX in different regions (cortex, hippocampus and cerebellum) of the brain from WT and rescued mice at 9 months old. Magnified are shown in square box.

B, **C**. Histological characterization of WWOX expression in tissues (Liver, Pancreas, Kidney Testis and ovary) of WT, KO, and KO+hWWOX at P17 (**B**) and 9 months old (**C**). Arrows in **B** indicates WWOX positivity in leydig cells. Representative images are shown from each genotype (*n*=3 per genotype).

Data information: Scale bars **A**) 100 µm (magnified 20 µm) and **B**) and **C**) 50 µm.

Appendix Fig S3. Evaluating motor coordination in *Wwox* null and rescued mice by performing hind-limb clasping test.

A-**C**. Images of hind-limb clasping test showing lack of coordination (closed hind legs- shown with red arrow) in *Wwox* null shown in **A**. After restoration of

WWOX in *Wwox* null by injecting AAV9-SynI-hWWOX virus, the motor coordination is restored in the rescued mice (stretched hind legs shown with purple arrows in two different mice) shown in **B** and **C** as compared to the WT.

Appendix Fig S4. Neuronal WWOX restoration improves the development of testis and bone.

A. Representative images showing the histology of testis from WT (*n*=4), KO (*n*=3), KO+AAV-mWwox (*n*=3) at P17. Arrows (black) indicate Leydig cells in WT, rescued mice and their absence in KO. Magnified area is shown with a box on the left panel.

B. Representative histological (longitudinal section) images of bone (tibia) from WT, KO, KO+AAV-mWwox at P17. Bar represents bone width from WT (156±13.8µm, *n*=3), KO (38.5±6.8µm, *n*=3) and KO+AAV-mWwox (130±7.9µm, *n*=3). Bone width was measured from 3 different regions of tibia from each mice (*n*=3) per genotype.

Data information: scale bars **A**) 50 µm **B**) 100 µm.

Appendix Fig S5. Surgical Procedures and Cell attached recordings of WT, KO and rescued (AAV9-mWwox or AAV9-hWWOX) mice.

A. A view of a juvenile mouse (P20) placed on the stereotax with an electrode inserted into the brain.

B. A 3-mm diameter craniotomy, positioned 1.6-2 mm posterior to the bregma and 4 mm lateral to the midline in a juvenile mouse.

C. A view of an adult mouse (6 months) placed on the stereotax with an electrode inserted into the brain.

D. A 3 mm diameter craniotomy, positioned 1.6-2 mm posterior to the bregma and 4 mm lateral to the midline in an adult mouse.

E. A biopsy punch was used to cut through

F. The electrodes were inserted at a 45 degrees and reached a depth of 200-300 µm.

G. Raster plots of cell-attached recordings (age 18-21) performed in WT (30 recorded neurons, 10 neurons from each animal, *n*=3), KO+A-mWwox (total 30, 10 neurons in each animal, *n*=3), KO+A-hWWOX (total 30, 10 neurons in each animal, *n*=3) and KO (total 45, 15 neurons in each animal, *n*=3). Each raster plot presents the results from one mouse. Every line within the raster plot presents one recorded neuron. The activity was recorded over 4 minutes and was binned at 2000 ms. The total amount of action potentials within the bin is color coded according to the color map presented. A clear hyperactivity was observed in the KO pups.

H. Raster plots of cell-attached recordings performed in adult (6 months) WT mice (*n*=3), KO+A-mWwox mice (*n*=3) and a total of 60 recorded neurons (20 neurons from each animal). Each raster plot presents the results from one mouse. Every line within the raster plot presents one recorded neuron. The activity was recorded over 4 minutes and was binned at 2000 ms. The total amount of action potentials within the bin is color coded according to the color map presented.

Appendix Fig S6. WWOX restoration in neurons (AAV9-hWWOX) improves myelination in *Wwox* null mice.

A. Images of brain sagittal sections that were immunolabelled with anti-MBP at P19 from WT, KO, KO+AAV9-hWWOX (*n*=3 for each group). Representative MBP staining (shown in gray) images from cortex (upper panel) and cerebellum (lower panel) are presented. Magnified regions marked with white boxes and their corresponding images are shown in right.

Data information: scale bars **A**) 100 µm.
