# Supplementary material for: Neonatal neuronal WWOX gene therapy rescues Wwox null phenotypes
Source: EMBO Mol Med. 2021 Nov 7;13(12):e14599. doi: 10.15252/emmm.202114599 (PMC8649866; doi:10.15252/emmm.202114599)
Supplement: Supplementary file 2 [file EMMM-13-e14599-s002.pdf]

EMBO Molecular Medicine

Neonatal neuronal WWOX gene therapy rescues Wwox null phenotypes

Srinivasa Repudi, Irina Kustanovich, Sara Abu-Swai, Shani Stern, and Rami Aqeilan
DOI: 10.15252/emmm.202114599

Corresponding author(s): Rami Aqeilan (ramiaq@mail.huji.ac.il)

Review Timeline:

Transfer from Review Commons:	24th May 21
Editorial Decision:	26th May 21
Revision Received:	14th Sep 21
Editorial Decision:	1st Oct 21
Revision Received:	14th Oct 21
Accepted:	15th Oct 21

Editor: Jingyi Hou

Transaction Report:

This manuscript was transferred to EMBO Molecular Medicine following peer review at Review Commons.

Review COMMONS

(Note: With the exception of the correction of typographical or spelling errors that could be a source of ambiguity, letters and reports are not edited. Depending on transfer agreements, referee reports obtained elsewhere may or may not be included in this compilation. Referee reports are anonymous unless the Referee chooses to sign their reports.)

26th May 2021

Dear Prof. Aqeilan,

Thank you for the submission of your manuscript to EMBO Molecular Medicine. I have now had a chance to carefully read your point-by-point response. I also discussed your work and your response to the referees' comments with other members of our editorial team. We think that the study is of potential interest and we would like to invite a major revision of your manuscript. Please also include the following points suggested by the referees in your revision:

- The expression pattern of the transgene in different brain regions and cell types needs to be better characterized (point 1 of Referee #2 and point 4 of Referee #3)
- 'n' needs to be increased wherever needed, as pointed out by Referees #2 and #3.
- A characterization of neuropathology is required (point #5 of Referee #3).

Further, given the relative preliminary nature of the presented findings, we think that additional experiments are needed to enhance the direct translational relevance of the study. In light of Referee #3's comments, the following issues need to be addressed:

- Data on long-term effects and transgene expression, as well as long-term toxicity following AAV administration, need to be provided.
- The referee's concern about the IRES-GFP sequence and the limitation in this regard need to be discussed. Still, we would strongly encourage you to include the data on plasmid without the IRES-GFP sequence.
- Testing alternative ROA is welcome but not required.
- Testing alternative vector is welcome but not mandatory.

Addressing the reviewers' concerns in full will be necessary for further considering the manuscript in our journal, and acceptance of the manuscript will entail a second round of review. EMBO Molecular Medicine encourages a single round of revision only. Therefore, acceptance or rejection of the manuscript will depend on the completeness of your responses included in the next, final version of the manuscript. For this reason, and to save you from any frustrations in the end, I would strongly advise against returning an incomplete revision.

We would welcome the submission of a revised version within three to six months for further consideration. However, we realize that the current situation is exceptional on account of the COVID-19/SARS-CoV-2 pandemic. Please let us know if you require longer to complete the revision.

Please read below for important editorial formatting and consult our author's guidelines for proper formatting of your revised article for EMBO Molecular Medicine.

I look forward to receiving your revised manuscript.

Sincerely,
Jingyi

Jingyi Hou
Editor
EMBO Molecular Medicine

*****

When submitting your revised manuscript, please carefully review the instructions that follow below. We perform an initial quality control of all revised manuscripts before re-review; failure to include requested items will delay the evaluation of your revision.

We require:

1) A .docx formatted version of the manuscript text (including legends for main figures, EV figures and tables). Please make sure that the changes are highlighted to be clearly visible.

2) Individual production quality figure files as .eps, .tif, .jpg (one file per figure). For guidance, download the 'Figure Guide PDF'

(https://www.embopress.org/page/journal/17574684/authorguide#figureformat).

3) A .docx formatted letter INCLUDING the reviewers' reports and your detailed point-by-point responses to their comments. As part of the EMBO Press transparent editorial process, the point-by-point response is part of the Review Process File (RPF), which will be published alongside your paper.

4) A complete author checklist, which you can download from our author guidelines (https://www.embopress.org/page/journal/17574684/authorguide#submissionofrevisions). Please insert information in the checklist that is also reflected in the manuscript. The completed author checklist will also be part of the RPF.

5) Please note that all corresponding authors are required to supply an ORCID ID for their name upon submission of a revised manuscript.

6) It is mandatory to include a 'Data Availability' section after the Materials and Methods. Before submitting your revision, primary datasets produced in this study need to be deposited in an appropriate public database, and the accession numbers and database listed under 'Data Availability'. Please remember to provide a reviewer password if the datasets are not yet public (see https://www.embopress.org/page/journal/17574684/authorguide#dataavailability).

In case you have no data that requires deposition in a public database, please state so in this section. Note that the Data Availability Section is restricted to new primary data that are part of this study.

7) For data quantification: please specify the name of the statistical test used to generate error bars and P values, the number (n) of independent experiments (specify technical or biological replicates) underlying each data point and the test used to calculate p-values in each figure legend. The figure legends should contain a basic description of n, P and the test applied. Graphs must include a description of the bars and the error bars (s.d., s.e.m.).

8) We would also encourage you to include the source data for figure panels that show essential data. Numerical data should be provided as individual .xls or .csv files (including a tab describing the data). For blots or microscopy, uncropped images should be submitted (using a zip archive if multiple images need to be supplied for one panel). Additional information on source data and instruction on how to label the files are available at

.

9) Our journal encourages inclusion of *data citations in the reference list* to directly cite datasets that were re-used and obtained from public databases. Data citations in the article text are distinct from normal bibliographical citations and should directly link to the database records from which the data can be accessed. In the main text, data citations are formatted as follows: "Data ref: Smith et al, 2001" or "Data ref: NCBI Sequence Read Archive PRJNA342805, 2017". In the Reference list, data citations must be labeled with "[DATASET]". A data reference must provide the database name, accession number/identifiers and a resolvable link to the landing page from which the data can be accessed at the end of the reference. Further instructions are available at .

10) We replaced Supplementary Information with Expanded View (EV) Figures and Tables that are collapsible/expandable online. A maximum of 5 EV Figures can be typeset. EV Figures should be cited as 'Figure EV1, Figure EV2" etc... in the text and their respective legends should be included in the main text after the legends of regular figures.

- For the figures that you do NOT wish to display as Expanded View figures, they should be bundled together with their legends in a single PDF file called *Appendix*, which should start with a short Table of Content. Appendix figures should be referred to in the main text as: "Appendix Figure S1, Appendix Figure S2" etc.

- Additional Tables/Datasets should be labeled and referred to as Table EV1, Dataset EV1, etc. Legends have to be provided in a separate tab in case of .xls files. Alternatively, the legend can be supplied as a separate text file (README) and zipped together with the Table/Dataset file.

See detailed instructions here:

.

11) The paper explained: EMBO Molecular Medicine articles are accompanied by a summary of the articles to emphasize the major findings in the paper and their medical implications for the non-specialist reader. Please provide a draft summary of your article highlighting

- the medical issue you are addressing,

- the results obtained and

- their clinical impact.

This may be edited to ensure that readers understand the significance and context of the research. Please refer to any of our published articles for an example.

12) For more information: There is space at the end of each article to list relevant web links for further consultation by our readers. Could you identify some relevant ones and provide such information as well? Some examples are patient associations, relevant databases, OMIM/proteins/genes links, author's websites, etc...

13) Author contributions: the contribution of every author must be detailed in a separate section (before the acknowledgments).

14) A Conflict of Interest statement should be provided in the main text.

15) Every published paper now includes a 'Synopsis' to further enhance discoverability. Synopses are displayed on the journal webpage and are freely accessible to all readers. They include a short stand first (maximum of 300 characters, including space) as well as 2-5 one-sentences bullet points that summarizes the paper. Please write the bullet points to summarize the key NEW findings. They should be designed to be complementary to the abstract - i.e. not repeat the same text. We encourage inclusion of key acronyms and quantitative information (maximum of 30 words / bullet point). Please use the passive voice. Please attach these in a separate file or send them by email, we will incorporate them accordingly.

Please also suggest a striking image or visual abstract to illustrate your article as a PNG file 550 px wide x 300-600 px high.

EMBO Molecular Medicine has a "scooping protection" policy, whereby similar findings that are published by others during review or revision are not a criterion for rejection. Should you decide to submit a revised version, I do ask that you get in touch after three months if you have not completed it, to update us on the status.

***** Reviewer's comments *****

***

Rev_Com_number: RC-2021-00783
New_manu_number: EMM-2021-14599
Corr_author: Aqeilan
Title: Neonatal neuronal WWOX gene therapy rescues Wwox null phenotypes

Review #1 -

This study clearly shows that neural Wwox gene therapy markedly improves most of phenotype exhibited in null Wwox mutation. This excellent study is supported by previous authors' work using neuron-specific knockout study. Purpose is clear, methods are reasonable, results are clearly shown, and discussions are well written. I have no doubt about this study.

This study is very important for future gene therapy of Wwox. It should be accepted for publication.

Review #2 -

In the manuscript entitled "Neonatal neuronal WWOX gene therapy rescues Wwox null phenotypes" by S. Repudi et al. report data on Wwox deficient mice treated with AAV vectors expressing either murine or human Wwox. The expression of Wwox was driven by the human Synapsin I promoter, mostly restricting the expression to neurons. The treatment was conducted at the day of birth by bi-hemispheric injection targeting the lateral ventricle. The authors report a marked rescue of the phenotype of Wwox null mice in a number of aspects. Overall, this proof of principle study suggests that early in life re-substitution of Wwox may represent a therapeutic option for the rare genetic disease caused by WWox loss of function in humans.
The data provided cover a broad spectrum of symptoms starting from survival rate over myelination to behavior. The data shown are convincing, although partially based on a very low n (some times only 2 animals).

Major comments:

1) The injection of the viral vector occurs under hard to control conditions. P0 pups are really small and 1 µl per hemisphere is a lot for them. Therefore, the expression pattern needs to be characterized better. The authors only controlled cortical tissue close to the injection site and claim CNS expression. However, there is no evidence for absence in the PNS and expression in brain regions distal to the injection.

2) Authors report rescue of growth and blood glucose levels in Wwox re-expressing mice. It would be interesting to see, whether this is solely due to involvement of Wwox in metabolic functions (presumably controlled by the CNS) or if motor impairment hinders the pups from normal feeding.

3) The evaluation of myelination and OPC differentiation as depicted in Fig. 4 a displays a clear improvement in MBP staining, yet KO+AAV-Wwox are still closer to KO than WT. This appears less pronounced in the Fig. 4 B and 4C with the CC1 staining. However, for the evaluation of cell numbers, tissue shrinkage and consequently overestimation of cell numbers / area have to be considered. How was this issue dealt with does not become clear from the methods section.

4) The authors claim reduced anxiety and improved motor coordination in Wwox deficient

mice treated with AAV vectors expressing either murine or human Wwox at the age of 8-10 weeks. However, Wwox deficient mice do not reach this age and therefore there is no control group demonstrating increased anxiety of impaired motor coordination. While a motor impairment appears very likely due to the neuropathological symptoms, increased anxiety cannot be given for granted.

5) In the behavioral experiments both sexes were tested, but not depicted for the OF test. However, the trace shown in Fig. 6 suggests a difference between WT and KO+AAV-Wwox. Overall, females are usually much more active in the OF, therefore combining the sexes increases variability. To reflect on trait anxiety, time/entries and distance traveled in the center as compared to the border have to be compared.

6) In several instances, only 2 mice were used. Although some of the experiments are technically challenging, a n of 3 is considered absolute minimum. The low n is mostly hidden by measuring several cells per animal, however the experimental animal has to be considered the biological replicate.

7) Statistical analysis and data presentation appear incorrect in several instances. Likewise mean {plus minus} SEM is given throughout, but in most instances mean {plus minus} SD would be appropriate. Also the two-tailed unpaired Student's t-test is not the correct choice in all instances. 2-way ANOVA would be needed for the behavioral test considering sex and treatment for example.

Minor comments

1) The information given in the method section doe snot always allow reproduction. Most obvious is the lack of information on antibodies used and their dilution. Moreover counting procedures for neuronal cell numbers needs better explanation: how were the counted areas defined? was CC counted over full width? was there some volumetry involved?

2) There is a difference between anticonvulsant and antiepileptic drugs, please check use of terms.

The significance for patients suffering from SCAR12 or WOREE might be high in case a gene therapy can be developed based on these data. However, the impact on the broader field is limited, considering the fact, that several studies before showed that rescue of phenotypes by re-substitution of a functional copy of a non-functional protein can be achieved by gene therapy. Actually such gene therapy is already in clinical use.

The audience will be mostly a very specialized group of scientists.

My field of expertise relevant for this manuscript covers immunohistochemistry and neuronal morphology, behavioral studies, CNS application of AAV vectors and in-vivo EEG.

Review #3 -

SUMMARY

The manuscript entitled 'Neonatal neuronal WWOX gene therapy rescues Wwox null phenotypes' describes a gene augmentation approach to treat the neurodegeneration associated with WWOX loss of function mutations. The authors designed an AAV9 vector expressing either the mouse or human WWOX gene, and delivered it intracerebroventricularly to neonatal Wwox KO mice. The treatment resulted in extended life span, improvement in neuronal activity, myelination and behaviour of treated mice.
Overall the study design is comprehensive and the authors have addressed different issues linked to the Wwox phenotype (myelination, motor coordination, glycaemic index).

COMMENTS:

1. P4: The authors delivered the vector to P0 mice, since the phenotype of the null mice is quite rapid and severe. They mention that Wwox KO in neuronal stem cell and progenitors result in the acute phenotype. Can the author elaborate further on how this translate to humans, and if a P0 injection could indeed target those progenitor cells?
2. P6: the antibody for WWOX seems to work quite well (fig 2). Can the authors elaborate on the reason to add the IRES-GFP sequence? This of course would have to be removed to perform clinically translatable experiments.
3. P6: the n number used in the hWWOX study is low, however it looks like treated animals started dying earlier than those treated with the mWwox vector. Can the authors speculate on this possible difference, given the high similarity of the two sequences?
4. P7, P13: hSyn promoter can be leaky and drive expression in other cell types (and organs) other than neurons. The authors should be careful to state that the effects of the treatment are caused by neuronal restoration only - no colocalization with other cell types have been performed. Can these affects caused by local AAV transduction?
5. The study would benefit from a neuropathology study, in order to characterise the brain pathology and further delineate the effect of the treatment on other cell types (ie is neuroinflammation present?). Also, it would be helpful to show IF staining of older mice brains.
6. P10: the authors do not clearly address the significant increase in unmyelinated axons in if 5E.
7. P10: could the authors comment on the sex difference in the rotarod test?
8. P19-20: do the authors have any behavioural data on older mice? Why were the tests not conducted on end-stage treated animals? were the animals trained at rotarod before testing? One experimental day without previous training might produce biased results.

MINOR COMMENTS

1. Although the majority of the study was conducted using the mWwox vector, also the human gene has been administered. Please state more clearly where mWwox was used in the figures and legends to avoid any confusion.
2. P8: Supplementary fig refers to S4.
3. P10: for technical reasons, the authors couldn't perform behavioural analysis on KO mice. Since no data is present for the null model, the authors shouldn't state the anxiety was reduced. This should be changed to comparative behaviour to WT.

4. P10: mention fig 6E.
5. P11: 'cDNA' in italic - change format.
6. P15: Please provide more accurate information on vector production manufacture and tittering method if available.
7. P17: please add antibody references and concentrations used.
8. P30: fig 1C,D please add p values.
9. P33: fig E repeated twice. Please change to F.
10. P33: please complete the sentence in fig S1.
11. P33: please add p values in fig S2.
12. P33: please add p values in fig S3.

SIGNIFICANCE

The work presented is novel in the field, as no gene therapy/gene delivery has been described in the literature so far. This approach has the potential to provide a treatment option for a devastating neurological condition, for which there is no available cure. The role of WWOX in several different pathways makes it an interesting target. At the same time, further research will be needed to elucidate the possible effects of the treatment on other brain regions or cellular pathways.

While this study has the potential to be translated to the clinic, further optimisation and testing will be needed. My expertise is gene delivery for the treatment of rare neurological disorders and viral vector manufacturing. From a gene therapy point of view, the study would have benefited from a more rational design of the experiments. For example: plasmid design (IRES-GFP is not needed. Why adding it?); or could a different ROA be explored - ie intraparenchymal? The authors do not address in detail which brain regions are particularly affected, and therefore it is difficult to predict if ICV is indeed the optimal ROA for this condition. Can a different or better vector be used? In addition, staining of brain sections from older mice would have helped to comment on long-term effect and transgene expression following AAV administration, which has not be addressed at all.

Point-by-point response

Reviewer #1 (Evidence, reproducibility and clarity (Required)):

This study clearly shows that neural Wwox gene therapy markedly improves most of phenotype exhibited in null Wwox mutation. This excellent study is supported by previous authors' work using neuron-specific knockout study. Purpose is clear, methods are reasonable, results are clearly shown, and discussions are well written. I have no doubt about this study.

Reviewer #1 (Significance (Required)):

This study is very important for future gene therapy of Wwox. It should be accepted for publication.

Response: We thank the esteemed reviewer for these comments.

Reviewer #2 (Evidence, reproducibility and clarity (Required)):

In the manuscript entitled "Neonatal neuronal WWOX gene therapy rescues Wwox null phenotypes" by S. Repudi et al. report data on Wwox deficient mice treated with AAV vectors expressing either murine or human Wwox. The expression of Wwox was driven by the human Synapsin I promoter, mostly restricting the expression to neurons. The treatment was conducted at the day of birth by bi-hemispheric injection targeting the lateral ventricle. The authors report a marked rescue of the phenotype of Wwox null mice in a number of aspects. Overall, this proof of principle study suggests that early in life re-substitution of Wwox may represent a therapeutic option for the rare genetic disease caused by WWox loss of function in humans.
The data provided cover a broad spectrum of symptoms starting from survival rate over myelination to behavior. The data shown are convincing, although partially based on a very low n (some times only 2 animals).

Response: We thank the esteemed reviewer for these comments and complements. All specific comments were considered and addressed as detailed below.

Major comments:

1) The injection of the viral vector occurs under hard to control conditions. P0 pups are really small and 1 µl per hemisphere is a lot for them. Therefore, the expression pattern needs to be characterized better. The authors only controlled cortical tissue close to the injection site and claim CNS expression. However,

there is no evidence for absence in the PNS and expression in brain regions distal to the injection.

Response: We thank the reviewer for this comment. We do agree that there might be inaccuracy in injecting the virus however this is a gold-standard method in neonatal mice. According to the literature (Kim et al., *EJN* 2013; Kim et al., *J Vis Exp* 2014; Durren et al., *Hu Gen The* 2014), it was shown to inject 2 µl per hemisphere to the neonatal mice, therefore we do not think that 1 µl volume is a lot to inject in these mice.

To address better characterization of expression of WWOX, we now provide transgene expression in several brain regions after treatment with AAV9-WWOX at juvenile and adult stages. These data are presented in new **Figure 1** & **2** and **EV2** and reveal prominent neuronal expression in all brain tissues. Quantification of WWOX expression in these brain regions is also included (**Fig 1E**, **Fig 2G**).

To address the leakiness of the vector upon intracerebrovetricular injection, we tested WWOX expression in peripheral tissues at P17 and 9-months old tissues and revealed no expression (**Fig EV2**).

Description of these results appears in page 7 & 8.

2) Authors report rescue of growth and blood glucose levels in Wwox re-expressing mice. It would be interesting to see, whether this is solely due to involvement of Wwox in metabolic functions (presumably controlled by the CNS) or if motor impairment hinders the pups from normal feeding.

Response: We thank the reviewer for this critical comment. In our recent paper in press (Repudi et al., *Brain* 2021), we documented that neuronal deletion of WWOX (ΔWWOX^{neurons}) recapitulates the Wwox null phenotype. Indeed, we found that these mice show hypoglycemia similar to the Wwox null, which indicates neuronal WWOX of the CNS regulates glucose levels in these mice and not the improper feeding by the mother as we could see milk in their stomach. In addition, we also tested keeping only Wwox null pup/pups with the mother to avoid the competition of their littermates for feeding but still these mice are hypoglycemic. Overall, neuronal WWOX restoration not only maintain the blood glucose levels but also improves the development of all other organs. The effect of neuronal WWOX on glucose homeostasis is very intriguing and will be subject of a separate study that we are conducting. We expanded this in the discussion, page 15, 2nd paragraph.

3) The evaluation of myelination and OPC differentiation as depicted in Fig. 4 a displays a clear improvement in MBP staining, yet KO+AAV-Wwox are still closer to KO than WT. This appears less pronounced in the Fig. 4 B and 4C with the CC1 staining. However, for the evaluation of cell numbers, tissue shrinkage and consequently overestimation of cell numbers / area have to be considered. How was this issue dealt with does not become clear from the methods section.

Response: We thank the reviewer for this important comment. Our data regarding improved myelination showed by MBP immunofluorescence staining in rescued mice are further supported by EM analysis (**Fig. 5**), representing the ultimate proof of WWOX effect on enhanced myelination. To further clarify the effect in MBP staining (**Fig 4A**), we drew a dotted line showing the boarder of the cortex highlighting the defect in KO and improved myelination in rescued mice. Further, in **Fig 4B**, we counted the number of CC1 and PDGFRα in corpus callosum in a $0.5mm^2$ (includes the marked area with a white dotted box) area from 3 identical sagittal sections from 3 different mice in each genotype. We explained this in **Fig. 4C** legend. We observed the significant increase in the number of CC1 positive cells in the rescued mice compared to the KO but in comparison to the WT there are less CC1 positive cells. We believe this could be related to oligodendrocyte-autonomous functions of WWOX. The same outcome and trend have been reproduced in sections obtained when rescuing with AAV9-SynI-hWWOX, presented in new **Fig EV1F**, showing increased CC1 staining.

4) The authors claim reduced anxiety and improved motor coordination in Wwox deficient mice treated with AAV vectors expressing either murine or human Wwox at the age of 8-10 weeks. However, Wwox deficient mice do not reach this age and therefore there is no control group demonstrating increased anxiety of impaired motor coordination. While a motor impairment appears very likely due to the neuropathological symptoms, increased anxiety cannot be given for granted.

Response: We thank the reviewer for this comment. It is difficult to check the anxiety in KO mice at 2-3 weeks of age, therefore we didn't consider these mice as our control group. We did these comparisons in adult mice but compared to WT mice, as KO die by this stage. We and authors (Cheng et al., *Acta Neuro Com* 2020) reported that the Wwox null mice show spontaneous seizures which presumably makes more anxiety. As we are unable to perform behavioral tests (open field and elevated plus maze) in KO mice, we increased our behavioral tests in as many as rescued mice as possible at different ages and tuned down our conclusion and indicated that restoration of WWOX is associated with normal behavior as WT mice (pages 12 & 13).

5) In the behavioral experiments both sexes were tested, but not depicted for the OF test. However, the trace shown in Fig. 6 suggests a difference between WT and KO+AAV-Wwox. Overall, females are usually much more active in the OF, therefore combining the sexes increases variability. To reflect on trait anxiety, time/entries and distance traveled in the center as compared to the border have to be compared.

Response: We thank the reviewer for this important comment. We agree and we will show males and females separately in the OF as suggested. Data is now provided per the reviewer request and appear in new **Figure 6**, described in page 12 and 13.

6) In several instances, only 2 mice were used. Although some of the

experiments are technically challenging, a n of 3 is considered absolute minimum. The low n is mostly hidden by measuring several cells per animal, however the experimental animal has to be considered the biological replicate.

Response: We thank the reviewer for this comment. All experiments are now containing at least n=3.

7) Statistical analysis and data presentation appear incorrect in several instances. Likewise mean {plus minus} SEM is given throughout, but in most instances mean {plus minus} SD would be appropriate. Also the two-tailed unpaired Student's t-test is not the correct choice in all instances. 2-way ANOVA would be needed for the behavioral test considering sex and treatment for example.

Response: We thank the reviewer for this important comment. We reorganized our data and analysis to show ±SD wherever is necessary. Behavioral tests data are presented now using 2-way ANOVA, as suggested.

Minor comments

1) The information given in the method section doesnot always allow reproduction. Most obvious is the lack of information on antibodies used and their dilution. Moreover counting procedures for neuronal cell numbers needs better explanation: how were the counted areas defined? was CC counted over full width? was there some volumetry involved?

Response: We thank the reviewer for this important comment. We apologize for not mentioning the information regarding antibodies, which we now added an appendix Table 1 with all relevant information. A note was added in the Methods' section in page 20.

We counted the number of CC1 and PDGFRα in corpus callosum in a 0.5mm^2 (includes the marked area with a white dotted box) area from 3 identical sagittal sections from 3 different mice in each genotype. We explained this in **Fig. 4C legend**.

2) There is a difference between anticonvulsant and antiepileptic drugs, please check use of terms.
Response: We thank the reviewer for this suggestion which we corrected in our revised manuscript.

Reviewer #2 (Significance (Required)):

The significance for patients suffering from SCAR12 or WOREE might be high in case a gene therapy can be developed based on these data. However, the impact on the broader field is limited, considering the fact, that several studies before showed that rescue of phenotypes by re-substitution of a functional copy of a non-functional protein can be achieved by gene therapy. Actually such gene

therapy is already in clinical use.

The audience will be mostly a very specialized group of scientists.

Response: We thank the reviewer for recognizing the significance of our work and that it lays the ground for clinical gene therapy trial to save WOREE and SACR12 children. WOREE syndrome is indeed a rare genetic disease affecting several hundreds of patients in the world in a given time. However, it is our duty as a biomedical researchers to help dissecting these maladies and provide answers and treatment options for the sick children and their suffering parents and families. The number of scientists working on these syndromes could also be limited and very specialized, but I think this proof of concept [PoC] should open more opportunities and help other researchers in exploring the strength of gene therapy in other epileptic encephalopathies and even other neurological diseases. Each gene therapy PoC is an achievement by itself and should give hope to the scientific community and the general public in curing fatal diseases such as WOREE and SCAR12 syndromes. I think it is our duty to promote excellence in translational medicine even though it deals with rare genetic diseases.

Reviewer #3 (Evidence, reproducibility and clarity (Required)):

SUMMARY

The manuscript entitled 'Neonatal neuronal WWOX gene therapy rescues Wwox null phenotypes' describes a gene augmentation approach to treat the neurodegeneration associated with WWOX loss of function mutations. The authors designed an AAV9 vector expressing either the mouse or human WWOX gene, and delivered it intracerebroventricularly to neonatal Wwox KO mice. The treatment resulted in extended life span, improvement in neuronal activity, myelination and behaviour of treated mice.
Overall the study design is comprehensive and the authors have addressed different issues linked to the Wwox phenotype (myelination, motor coordination, glycaemic index).

Response: We thank the reviewer for acknowledging the importance of our study.

COMMENTS:

1. P4: The authors delivered the vector to P0 mice, since the phenotype of the null mice is quite rapid and severe. They mention that Wwox KO in neuronal stem cell and progenitors result in the acute phenotype. Can the author elaborate

further on how this translate to humans, and if a P0 injection could indeed target those progenitor cells?

Response: We thank the reviewer for this comment. In our recent paper (Repudi et al., *Brain* 2021), we documented the conditional ablation in Neural stem and progenitor cells ($\Delta WWOX^{NSPS}$ or N-KO) reproduces the Wwox null phenotype. Since Neural stem and progenitor cells differentiate into other cell types, we conditionally deleted Wwox in neurons (using Synapsin Cre), astrocytes (GFAP-Cre) OPCs and OLs (Olig2- Cre). Remarkably, conditional deletion of Wwox especially in neurons recapitulate the N-KO and Wwox null. This led us to propose restoring WWOX in neurons by selective use of the synapsin promoter using AAV. As our results show remarkable outcome, we believe that neuronal WWOX is responsible for the phenotype. AAV infect non-dividing cells and should retain expression for extended time. Since, we are using neuronal specific promoter, we do not expect to see the WWOX expression in progenitor cells. Our proof-of-concept results and outcomes also suggest that treating young children with AAV9-Synapsin-WWOX, which should infect mature neurons, has the potential to work and have an effect.

In the result section in page 5 and 6, we referred to this and indicated that "*Successful delivery of AAV9-hSynI-mWwox-IRES-EGFP (AAV-mWwox) or AAV9-hSynI-hWWOX (AAV-hWWOX) should lead to expression of intact WWOX protein in Synapsin-I-positive non-dividing/matured neurons.".*

2. P6: the antibody for WWOX seems to work quite well (fig 2). Can the authors elaborate on the reason to add the IRES-GFP sequence? This of course would have to be removed to perform clinically translatable experiments.

Response: Initially we chose to have the GFP reporter in our murine vector to check the expression pattern and ensure delivery to expected regions. In our humanized vector, which we refer to as AAV9-hWWOX, we removed the IRES-GFP sequence and examined efficacy and showed similar outcome as AAV9-mWwox. Validation of AAV9-hWWOX appears in new **EV1**, **Figure 2** and results were explained in pages 6-8.

Essentially, it should be noted that our analyses didn't reveal any difference between mWwox and hWWOX vectors.

3. P6: the n number used in the hWWOX study is low, however it looks like treated animals started dying earlier than those treated with the mWwox vector. Can the authors speculate on this possible difference, given the high similarity of the two sequences?

Response: We thank the reviewer for this observation. We made more injections of hWWOX and updated our *n* in all experiments as appear in our revised

version. The results are consistent and provide statistical significance as indicated in the different panels and new figures throughout the manuscript.

4. P7, P13: hSyn promoter can be leaky and drive expression in other cell types (and organs) other than neurons. The authors should be careful to state that the effects of the treatment are caused by neuronal restoration only - no colocalization with other cell types have been performed. Can these affects caused by local AAV transduction?

Response: We thank the reviewer for this important comment as promoters could be indeed leaky. In our revised submission, we provide evidence that WWOX restoration takes place only in neurons and not in oligodendrocytes by co-staining either with NeuN or CC1 antibodies along with anti-WWOX (shown in **Fig 1D, EV1E, EV1F and Fig 2D-F**). We have also tested the leakiness issue and reveal lack of expression in peripheral tissues as demonstrated in new **Fig EV2B** & **C**. Description of these results appears in pages 7 & 8.

5. The study would benefit from a neuropathology study, in order to characterise the brain pathology and further delineate the effect of the treatment on other cell types (ie is neuroinflammation present?). Also, it would be helpful to show IF staining of older mice brains.

Response: We thank the reviewer for this important comment. To address this comment, we immunostaied for astrocyte [GFAP] and microglial [Iba1] markers in brain of juvenile and adult treated mice. The results are described in page 11 [1st paragraph] and appear in new **Figure 3E-J**.

6. P10: the authors do not clearly address the significant increase in unmyelinated axons in if 5E.

Response: We thank the reviewer for this comment. The slight increase in unmyelinated axons could be due to some cell autonomous function of WWOX in OPCs differentiation in aged mice, which is supported by loss/ decreased levels of WWOX associated with age related neurological disorder like multiple sclerosis. We added a note in the result section in page 12 and in the discussion in page 16 of the revised manuscript.

7. P10: could the authors comment on the sex difference in the rotarod test?

Response: Although we didn’t observe significant changes between the different sexes we are now showing separate sexes in new **figure 6**.

8. P19-20: do the authors have any behavioural data on older mice? Why were the tests not conducted on end-stage treated animals? were the animals trained at rotarod before testing? One experimental day without previous training might produce biased results.

Response: We thank the reviewer for this comment. We now performed additional behavioral tests on additional number of mice at different stages that appear in New **Figure 6**. Importantly, we don’t observe significant differences between WT and rescued mice.
MINOR COMMENTS

1. Although the majority of the study was conducted using the mWwox vector, also the human gene has been administered. Please state more clearly where mWwox was used in the figures and legends to avoid any confusion.
2. P8: Supplementary fig refers to S4.
3. P10: for technical reasons, the authors couldn't perform behavioural analysis on KO mice. Since no data is present for the null model, the authors shouldn't state the anxiety was reduced. This should be changed to comparative behaviour to WT.
4. P10: mention fig 6E.
5. P11: 'cDNA' in italic - change format.
6. P15: Please provide more accurate information on vector production manufacture and tittering method if available.
7. P17: please add antibody references and concentrations used.
8. P30: fig 1C,D please add p values.
9. P33: fig E repeated twice. Please change to F.
10. P33: please complete the sentence in fig S1.
11. P33: please add p values in fig S2.
12. P33: please add p values in fig S3.

Response: We thank the reviewer for all these minor comments, all changes were considered and corrected.

Reviewer #3 (Significance (Required)):

SIGNIFICANCE

The work presented is novel in the field, as no gene therapy/gene delivery has been described in the literature so far. This approach has the potential to provide a treatment option for a devastating neurological condition, for which there is no available cure. The role of WWOX in several different pathways makes it an interesting target. At the same time, further research will be needed to elucidate the possible effects of the treatment on other brain regions or cellular pathways.

While this study has the potential to be translated to the clinic, further optimisation and testing will be needed. My expertise is gene delivery for the treatment of rare neurological disorders and viral vector manufacturing. From a gene therapy point of view, the study would have benefited from a more rational design of the experiments. For example: plasmid design (IRES-GFP is not needed. Why adding it?); or could a different ROA be explored - ie intraparenchymal? The authors do not address in detail which brain regions are particularly affected, and therefore it is difficult to predict if ICV is indeed the

optimal ROA for this condition. Can a different or better vector be used? In addition, staining of brain sections from older mice would have helped to comment on long-term effect and transgene expression following AAV administration, which has not been addressed at all.

Response: We thank the reviewer for acknowledging the novelty and significance of our study and its potential for advancing the treatment for WOREE children. In our revised manuscript we introduced our humanized vector using hWWOX and no GFP reporter or any additional sequence or elements that are not needed or allowed for human treatment. The initial use of murine and GFP reporter was for the proof-of-concept of our findings. It should be noted however that we observed no differences in efficacy when using mWwox or hWWOX sequence in our vectors.

There is certainly also much work to be done in the direction of cellular and molecular aspects and this is being done in parallel. Regarding the ROA, injecting into intracerebroventricular region of neonates has been shown to have widespread expression of the transgene (Kim et al., *EJN* 2013; Kim et al., *J Vis Exp* 2014; Durren et al., *Hu Gen The* 2014). Technically, intraparenchymal injection to the neonates in rodents are difficult. This route could be much easier in human patients. Intraperitoneal injection requires a lot of the virus and is not feasible for human trials. Intravenous injections were explored but due to the poor conditions of the null mice we could not perform. We are currently exploring different ROA to fit for older pups, but this is beyond the scope of our current study. Our study here aimed to show efficacy for neonatal and provide proof-of-concept which we did and with remarkable outcome we would like to move forward. As for vector choice, we chose AAV9 as it is in clinical use for treating SMA produced by Avexis as Zolgensma (Mendell et al., *NEJM* 2017) and again this is proof-of-concept. A note in the discussion was made about these issues in page 17.

1st Oct 2021

Dear Prof. Aqeilan,

Thank you for the submission of your revised manuscript to EMBO Molecular Medicine. We have now received the enclosed report from the two referees who were asked to re-assess it. As you will see the referees are now overall supportive and I am pleased to inform you that we will be able to accept your manuscript pending the following amendments:

1. Please address the remaining concerns from Referee #2 in writing.

2. In the main manuscript file, please do the following:
- Reduce keyword number to 5.
- Remove "data not shown". As per our guidelines, on "Unpublished Data" the journal does not permit citation of "data not shown". All data referred to in the paper should be displayed in the main or Expanded View figures.
- FIGURE CALLOUTS: missing 1F, 6A. Please fix this.
- in Materials and Methods, for animal work, gender, age, and genetic background must be indicated, along with housing conditions.

3. Please combine the Appendix Table 1 with 5 EV Figures (and their legends) into one single pdf called "Appendix".
- Appendix Table 1 needs to be renamed to "Appendix Table S1". Please update the callouts in the main text accordingly.
- EV figures need to be renamed to "Appendix Figure Sx". Please update all the callouts in the main text and make sure all the Appendix Figures are called for.
- Please remove the EV figure legend from the main manuscript text.
- Add a Table of Content on the 1st page.
- Please note that this file will not be typeset nor proofread.

4. Data availability: Please add a "Data availability" section. Since this study does not generate large-scale datasets, please only include the following sentence in this section- "This study includes no data deposited in external repositories".

5. Please provide a 'Synopsis' to further enhance discoverability. Synopses are displayed on the journal webpage and are freely accessible to all readers. They include a short stand first (maximum of 300 characters, including space) as well as 2-5 one-sentence bullet points that summarise the paper. Please write the bullet points to summarise the key NEW findings. They should be designed to be complementary to the abstract - i.e. not repeat the same text. We encourage inclusion of key acronyms and quantitative information (maximum of 30 words / bullet point). Please use the passive voice. Please attach these in a separate file or send them by email, we will incorporate them accordingly.

6. I have resized the synopsis image to the required 550-pixel width (see attached). Can you please confirm whether you are fine with the resized image? Please note that this would be the final version, and changes during proofing are usually not allowed.

7. Our data editors have seen the manuscript, and they have made some comments and suggestions that need to be addressed (see attached). Please send back a revised version (in track change mode), as we will need to go through the changes.

8. As part of the EMBO Publications transparent editorial process initiative (see our Editorial at http://embomolmed.embopress.org/content/2/9/329), EMBO Molecular Medicine will publish online a Review Process File (RPF) to accompany accepted manuscripts.

a. In the event of acceptance, this file will be published in conjunction with your paper and will include the anonymous referee reports, your point-by-point response and all pertinent correspondence relating to the manuscript. Let us know if you do not agree with this.

b. Please note that the Authors checklist will be published at the end of the RPF.

I look forward to receiving your revised manuscript soon.

Kind regards,
Jingyi

Jingyi Hou
Editor
EMBO Molecular Medicine

*** Instructions to submit your revised manuscript ***

*** PLEASE NOTE *** As part of the EMBO Publications transparent editorial process initiative (see our Editorial at https://www.embopress.org/doi/pdf/10.1002/emmm.201000094), EMBO Molecular Medicine will publish online a Review Process File to accompany accepted manuscripts.

In the event of acceptance, this file will be published in conjunction with your paper and will include the anonymous referee reports, your point-by-point response and all pertinent correspondence relating to the manuscript. If you do NOT want this file to be published, please inform the editorial office at contact@embomolmed.org.

To submit your manuscript, please follow this link:

https://embomolmed.msubmit.net/cgi-bin/main.plex

When submitting your revised manuscript, please include:

1) a .docx formatted version of the manuscript text (including Figure legends and tables)

2) Separate figure files*

3) supplemental information as Expanded View and/or Appendix. Please carefully check the authors guidelines for formatting Expanded view and Appendix figures and tables at https://www.embopress.org/page/journal/17574684/authorguide#expandedview

4) a letter INCLUDING the reviewer's reports and your detailed responses to their comments (as Word file).

5) The paper explained: EMBO Molecular Medicine articles are accompanied by a summary of the articles to emphasize the major findings in the paper and their medical implications for the non-specialist reader. Please provide a draft summary of your article highlighting
- the medical issue you are addressing,
- the results obtained and
- their clinical impact.
This may be edited to ensure that readers understand the significance and context of the research.
Please refer to any of our published articles for an example.

6) For more information: There is space at the end of each article to list relevant web links for further consultation by our readers. Could you identify some relevant ones and provide such information as well? Some examples are patient associations, relevant databases, OMIM/proteins/genes links, author's websites, etc...

7) Author contributions: the contribution of every author must be detailed in a separate section.

8) EMBO Molecular Medicine now requires a complete author checklist (https://www.embopress.org/page/journal/17574684/authorguide) to be submitted with all revised manuscripts. Please use the checklist as guideline for the sort of information we need WITHIN the manuscript. The checklist should only be filled with page numbers were the information can be found. This is particularly important for animal reporting, antibody dilutions (missing) and exact values and n that should be indicted instead of a range.

9) Every published paper now includes a 'Synopsis' to further enhance discoverability. Synopses are displayed on the journal webpage and are freely accessible to all readers. They include a short stand first (maximum of 300 characters, including space) as well as 2-5 one sentence bullet points that summarise the paper. Please write the bullet points to summarise the key NEW findings. They should be designed to be complementary to the abstract - i.e. not repeat the same text. We encourage inclusion of key acronyms and quantitative information (maximum of 30 words / bullet point). Please use the passive voice. Please attach these in a separate file or send them by email, we will incorporate them accordingly.

You are also welcome to suggest a striking image or visual abstract to illustrate your article. If you do please provide a jpeg file

550 px-wide x 400-px high.

10) A Conflict of Interest statement should be provided in the main text

11) Please note that we now mandate that all corresponding authors list an ORCID digital identifier. This takes <90 seconds to complete. We encourage all authors to supply an ORCID identifier, which will be linked to their name for unambiguous name identification.

Currently, our records indicate that the ORCID for your account is 0000-0002-6034-023X.

Please click the link below to modify this ORCID:
Link Not Available

12) The system will prompt you to fill in your funding and payment information. This will allow Wiley to send you a quote for the article processing charge (APC) in case of acceptance. This quote takes into account any reduction or fee waivers that you may be eligible for. Authors do not need to pay any fees before their manuscript is accepted and transferred to our publisher.

*Additional important information regarding Figures

Each figure should be given in a separate file and should have the following resolution:
Graphs 800-1,200 DPI
Photos 400-800 DPI
Colour (only CMYK) 300-400 DPI"

Figures are not edited by the production team. All lettering should be the same size and style; figure panels should be indicated by capital letters (A, B, C etc). Gridlines are not allowed except for log plots. Figures should be numbered in the order of their appearance in the text with Arabic numerals. Each Figure must have a separate legend and a caption is needed for each panel.

*Additional important information regarding figures and illustrations can be found at https://bit.ly/EMBOPressFigurePreparationGuideline. See also figure legend preparation guidelines: https://www.embopress.org/page/journal/17574684/authorguide#figureformat

The system will prompt you to fill in your funding and payment information. This will allow Wiley to send you a quote for the article processing charge (APC) in case of acceptance. This quote takes into account any reduction or fee waivers that you may be eligible for. Authors do not need to pay any fees before their manuscript is accepted and transferred to our publisher.

***** Reviewer's comments *****

Referee #1 (Comments on Novelty/Model System for Author):

This is a straight forward study on the rescue of the phenotype of a WWOX deficient mouse line as a proof of concept for a gene therapy. The use of the model and the use of 2 vectors, one more experimental (mWWOX & GFP) and the other already more clinical (hWWOX) is a good choice for this.

Referee #1 (Remarks for Author):

The revised version of the manuscript entitled "Neonatal neuronal WWOX gene therapy rescues Wwox null phenotypes" by S. Repudi et al. now addressed most of my concerns. It reads very well and provides clear and broad proof of concept.

Open points:

@ previous 2) I still do not agree, that normal blood glucose levels can be attributed to neuronal homeostasis from present data. WWOX KO display ataxia and therefore might be not able to eat properly in a normal cage. Similar effects have been observed in mGluR1 KO mice. They are also ataxic, but grew to almost normal weight when fed with ground chow in the cage. The argument with milk in the stomach does not address this point. If you look on the weight development, this appears fairly normal in KO mice as long as mice drink, but starts to be reduced when pups start to eat chow.

@ previous 3) a clear description of stereological measures is still missing in the methods section.

@ previous 4) The title of the chapter is still misleading, as it is still stated in the last sentence, that the treatment restores deficits of KO mice. As there is no KO control, this cannot be stated. Simply state that there is no difference to WT mice and the statements are correct.

In addition, I think it would be fair to mention the increased number of unmyelinated axons also in the results text.

Referee #2 (Comments on Novelty/Model System for Author):

Authors addressed most of the comments, adding suitable data and reviewing thoroughly the manuscript.

Point-by-point addressing Editor's and Reviewers' comments:

Editor's comments:

1. Please address the remaining concerns from Referee #2 in writing.

Response: Done, see point-by-point.

2. In the main manuscript file, please do the following:

- Reduce keyword number to 5.

Response: Done

- Remove "data not shown". As per our guidelines, on "Unpublished Data" the journal does not permit citation of "data not shown". All data referred to in the paper should be displayed in the main or Expanded View figures.

*Response: removed and added new figures (**Fig 4 G-I** and **Appendix Fig S6**).*

- FIGURE CALLOUTS: missing 1F, 6A. Please fix this.

Response: Done

- in Materials and Methods, for animal work, gender, age, and genetic background must be indicated, along with housing conditions.

Response: Done

3. Please combine the Appendix Table 1 with 5 EV Figures (and their legends) into one single pdf called "Appendix".

- Appendix Table 1 needs to be renamed to "Appendix Table S1". Please update the callouts in the main text accordingly.

- EV figures need to be renamed to "Appendix Figure Sx". Please update all the callouts in the main text and make sure all the Appendix Figures are called for.

- Please remove the EV figure legend from the main manuscript text.

- Add a Table of Content on the 1st page. - Please note that this file will not be typeset nor proofread.

Response: Done

4. Data availability: Please add a "Data availability" section. Since this study does not generate large-scale datasets, please only include the following sentence in this section- "This study includes no data deposited in external repositories".

Response: Done

5. Please provide a 'Synopsis' to further enhance discoverability. Synopses are displayed on the journal webpage and are freely accessible to all readers. They include a short stand first (maximum of 300 characters, including space) as well as 2-5 one-sentence bullet points that summarise the paper. Please write the bullet points to summarise the key NEW findings. They should be designed to be complementary to the abstract - i.e. not repeat the same text. We encourage inclusion of key acronyms and quantitative information (maximum of 30 words / bullet point). Please use the passive voice. Please attach these in a separate file or send them by email, we will incorporate them accordingly.

Response: Added Synopsis.

6. I have resized the synopsis image to the required 550-pixel width (see attached). Can you please confirm whether you are fine with the resized image? Please note that this would be the final version, and changes during proofing are usually not allowed.

Response: OK.

7. Our data editors have seen the manuscript, and they have made some comments and suggestions that need to be addressed (see attached). Please send back a revised version (in track change mode), as we will need to go through the changes.

Response: Submitted with Track-changes

8. As part of the EMBO Publications transparent editorial process initiative (see our Editorial at http://embomolmed.embopress.org/content/2/9/329), EMBO Molecular Medicine will publish online a Review Process File (RPF) to accompany accepted manuscripts.

a. In the event of acceptance, this file will be published in conjunction with your paper and will include the anonymous referee reports, your point-by-point response and all pertinent correspondence relating to the manuscript. Let us know if you do not agree with this.

b. Please note that the Authors checklist will be published at the end of the RPF. I look forward to receiving your revised manuscript soon.

Response: OK

Reviewer's comments

Referee #1 (Comments on Novelty/Model System for Author):

This is a straight forward study on the rescue of the phenotype of a WWOX deficient mouse line as a proof of concept for a gene therapy. The use of the model and the use of 2 vectors, one more experimental (mWWOX & GFP) and the other already more clinical (hWWOX) is a good choice for this.

Referee #1 (Remarks for Author): The revised version of the manuscript entitled "Neonatal neuronal WWOX gene therapy rescues Wwox null phenotypes" by S. Repudi et al. now addressed most of my concerns. It reads very well and provides clear and broad proof of concept.

***Response**: We thank the esteemed reviewer for these comments and appreciation.*

Open points:

@ previous 2) I still do not agree, that normal blood glucose levels can be attributed to neuronal homeostasis from present data. WWOX KO display ataxia and therefore might be not able to eat properly in a normal cage. Similar effects have been observed in mGluR1 KO mice. They are also ataxic, but grew to almost normal weight when fed with ground chow in the cage. The argument with milk in the stomach does not address this point. If you look on the weight development, this appears fairly normal in KO mice as long as mice drink, but starts to be reduced when pups start to eat chow.

Response: We thank the reviewer for this comment. In a separate study, we did try putting wet food (soaked with water) or high fat diet (HFD) starting from P7 on a plate in the cage (without having any other litter) so that the KO pups could easily eat, but still gradually these KO mice are deteriorating and eventually die. It is well known that Insulin Growth Factor 1 (IGF1) is majorly produced in the liver, and is stimulated by GH (Growth Hormone) that is regulated by hypothalamic neuronal stimulus from the brain. Unpublished observations from our lab show that Wwox-KO mice have less IGF-1 levels in their serum and in the liver. Therefore, we strongly believe that WWOX neuronal restoration of could reverse the hypoglycemic effect partly by regulating the hypothalamic- GH-IGF1 axis. We are working to further study this axis in Wwox-KO mice.

@ previous 3) a clear description of stereological measures is still missing in the methods section.

Response: Description of the stereological measures are provided in the materials and methods section under “cell attached recordings” and states:

“The electrode was inserted at a 45 degrees angle and reached a depth of 300 μm. The electrode positioning was targeted on the brain surface, positioned at 1.6-2mm posterior to the bregma and 4 mm lateral to the midline. While positioning the electrode, an increase of the

pipette resistance to 10–200 MOhm resulted in most cases in the appearance of action potentials (spikes). The detection of a single spike was the criteria to start the recording".

@ previous 4) The title of the chapter is still misleading, as it is still stated in the last sentence, that the treatment restores deficits of KO mice. As there is no KO control, this cannot be stated. Simply state that there is no difference to WT mice and the statements are correct.

Response: *We thank the reviewer for these suggestions. We now changed the sub-titles in results section.*

In addition, I think it would be fair to mention the increased number of unmyelinated axons also in the results text.

Response: *Increased number of unmyelinated axons was added in the text as suggested.*

Referee #2 (Comments on Novelty/Model System for Author):

Authors addressed most of the comments, adding suitable data and reviewing thoroughly the manuscript.

Response: *We thank the esteemed reviewer.*

15th Oct 2021

Dear Prof. Aqeilan,

We are pleased to inform you that your manuscript is accepted for publication and is now being sent to our publisher to be included in the next available issue of EMBO Molecular Medicine.

We would like to remind you that as part of the EMBO Publications transparent editorial process initiative, EMBO Molecular Medicine will publish a Review Process File online to accompany accepted manuscripts. If you do NOT want the file to be published or would like to exclude figures, please immediately inform the editorial office via e-mail.

Please read below for additional IMPORTANT information regarding your article, its publication and the production process.

Congratulations on your interesting work,
Jingyi

Jingyi Hou
Editor
EMBO Molecular Medicine

Follow us on Twitter @EmboMolMed
Sign up for eTOCs at embopress.org/alertsfeeds

*** *** *** IMPORTANT INFORMATION *** *** ***

SPEED OF PUBLICATION
The journal aims for rapid publication of papers, using using the advance online publication "Early View" to expedite the process: A properly copy-edited and formatted version will be published as "Early View" after the proofs have been corrected. Please help the Editors and publisher avoid delays by providing e-mail address(es), telephone and fax numbers at which author(s) can be contacted.

Should you be planning a Press Release on your article, please get in contact with embomolmed@wiley.com as early as possible, in order to coordinate publication and release dates.

LICENSE AND PAYMENT:

All articles published in EMBO Molecular Medicine are fully open access: immediately and freely available to read, download and share.

EMBO Molecular Medicine charges an article processing charge (APC) to cover the publication costs. You, as the corresponding author for this manuscript, should have already received a quote with the article processing fee separately. Please let us know in case this quote has not been received.

Once your article is at Wiley for editorial production you will receive an email from Wiley's Author Services system, which will ask you to log in and will present you with the publication license form for completion. Within the same system the publication fee can be paid by credit card, an invoice, pro forma invoice or purchase order can be requested.

Payment of the publication charge and the signed Open Access Agreement form must be received before the article can be published online.

PROOFS

You will receive the proofs by e-mail approximately 2 weeks after all relevant files have been sent o our Production Office. Please return them within 48 hours and if there should be any problems, please contact the production office at embopressproduction@wiley.com.

Please inform us if there is likely to be any difficulty in reaching you at the above address at that time. Failure to meet our deadlines may result in a delay of publication.

All further communications concerning your paper proofs should quote reference number EMM-2021-14599-V3 and be directed to the production office at embopressproduction@wiley.com.

Thank you,

Jingyi Hou
Editor
EMBO Molecular Medicine

***
Rev_Com_number: RC-2021-00783
New_manu_number: EMM-2021-14599-V3
Corr_author: Aqeilan
Title: Neonatal neuronal WWOX gene therapy rescues Wwox null phenotypes

EMBO PRESS

YOU MUST COMPLETE ALL CELLS WITH A PINK BACKGROUND ↓

PLEASE NOTE THAT THIS CHECKLIST WILL BE PUBLISHED ALONGSIDE YOUR PAPER

Corresponding Author Name: Rami I. Aqeilan
Journal Submitted to: EMBO Molecular Medicine
Manuscript Number: EMM-2021-14599-V2

Reporting Checklist For Life Sciences Articles (Rev. June 2017)

This checklist is used to ensure good reporting standards and to improve the reproducibility of published results. These guidelines are consistent with the Principles and Guidelines for Reporting Preclinical Research issued by the NIH in 2014. Please follow the journal's authorship guidelines in preparing your manuscript.

A- Figures
1. Data
The data shown in figures should satisfy the following conditions:

- the data were obtained and processed according to the field's best practice and are presented to reflect the results of the experiments in an accurate and unbiased manner.
- figure panels include only data points, measurements or observations that can be compared to each other in a scientifically meaningful way.
- graphs include clearly labeled error bars for independent experiments and sample sizes. Unless justified, error bars should not be shown for technical replicates.
- if n< 5, the individual data points from each experiment should be plotted and any statistical test employed should be justified
- Source Data should be included to report the data underlying graphs. Please follow the guidelines set out in the author ship guidelines on Data Presentation.

2. Captions
Each figure caption should contain the following information, for each panel where they are relevant:

- a specification of the experimental system investigated (eg cell line, species name).
- the assay(s) and method(s) used to carry out the reported observations and measurements
- an explicit mention of the biological and chemical entity(ies) that are being measured.
- an explicit mention of the biological and chemical entity(ies) that are altered/varied/perturbed in a controlled manner.

- the exact sample size (n) for each experimental group/condition, given as a number, not a range;
- a description of the sample collection allowing the reader to understand whether the samples represent technical or biological replicates (including how many animals, litters, cultures, etc.).
- a statement of how many times the experiment shown was independently replicated in the laboratory.
- definitions of statistical methods and measures:
  - common tests, such as t-test (please specify whether paired vs. unpaired), simple χ^2 tests, Wilcoxon and Mann-Whitney tests, can be unambiguously identified by name only, but more complex techniques should be described in the methods section;
  - are tests one-sided or two-sided?
  - are there adjustments for multiple comparisons?
  - exact statistical test results, e.g., P values = x but not P values < x;
  - definition of 'center values' as median or average;
  - definition of error bars as s.d. or s.e.m.

Any descriptions too long for the figure legend should be included in the methods section and/or with the source data.

In the pink boxes below, please ensure that the answers to the following questions are reported in the manuscript itself. Every question should be answered. If the question is not relevant to your research, please write NA (non applicable). We encourage you to include a specific subsection in the methods section for statistics, reagents, animal models and human subjects.

USEFUL LINKS FOR COMPLETING THIS FORM

http://www.antibodypedia.com
http://1degreebio.org
http://www.equator-network.org/reporting-guidelines/improving-bioscience-research-repor
http://grants.nih.gov/grants/olaw/olaw.htm
http://www.mrc.ac.uk/Ourresearch/Ethicsresearchguidance/Useofanimals/index.htm
http://ClinicalTrials.gov
http://www.consort-statement.org
http://www.consort-statement.org/checklists/view/32-consort/66-title
http://www.equator-network.org/reporting-guidelines/reporting-recommendations-for-tumo
http://datadryad.org
http://figshare.com
http://www.ncbi.nlm.nih.gov/gap
http://www.ebi.ac.uk/ega
http://biomodels.net/
http://biomodels.net/miriam/
http://jjj.biochem.sun.ac.za
http://oba.od.nih.gov/biosecurity/biosecurity_documents.html
http://www.selectagents.gov/

B- Statistics and general methods

Please fill out these boxes ↓ (Do not worry if you cannot see all your text once you press return)

1.a. How was the sample size chosen to ensure adequate power to detect a pre-specified effect size?	Sample size was chosen empiraclly, according to the accepted sample sizes used in the litrature of the field. At least n=3 were used all experiments.
1.b. For animal studies, include a statement about sample size estimate even if no statistical methods were used.	For most animal studeis, n was at least 3 for most experiment.
2. Describe inclusion/exclusion criteria if samples or animals were excluded from the analysis. Were the criteria pre-established?	Generally, no animals were excluded from analysis.
3. Were any steps taken to minimize the effects of subjective bias when allocating animals/samples to treatment (e.g. randomization procedure)? If yes, please describe.	NA
For animal studies, include a statement about randomization even if no randomization was used.	No randomization was applied here
4.a. Were any steps taken to minimize the effects of subjective bias during group allocation or/and when assessing results (e.g. blinding of the investigator)? If yes please describe.	The data was analyzed by the researchers themselves, therefore this was not a blind study. For assays that allow only a display of a representative image, typically a quantification was preformed, allowing for the assesments of multiple biological replicates. Furthermore, each assay was typically supported by alternative approaches, ideally ones that asses the organoids as a whole (e.g. Western blot/ qPCR analysis to support IF staining).
4.b. For animal studies, include a statement about blinding even if no blinding was done	The data was analyzed by the researchers themselves, therefore this was not a blind study.
5. For every figure, are statistical tests justified as appropriate?	Yes. Data are presented as either mean ± Standard deviation (SD) or standard Error of the Mean (SEM) or Anova, as indicated in the figure legend. P-values are presented in each figure using asterisks as indicated in the figure legened as well.
Do the data meet the assumptions of the tests (e.g., normal distribution)? Describe any methods used to assess it.	The two-tailed unpaired Student's t-test or Two-way ANOVA with Benforroni for post hoc comparisons was used to calculate the significance
Is there an estimate of variation within each group of data?	No.

Is the variance similar between the groups that are being statistically compared?	The varaicne was not fornally tested.

C- Reagents

6. To show that antibodies were profiled for use in the system under study (assay and species), provide a citation, catalog number and/or clone number, supplementary information or reference to an antibody validation profile. e.g., Antibodypedia (see link list at top right), 1DegreeBio (see link list at top right).	Please refer to Appendix Table 1 - Antibodies.
7. Identify the source of cell lines and report if they were recently authenticated (e.g., by STR profiling) and tested for mycoplasma contamination.	NA

* for all hyperlinks, please see the table at the top right of the document

D- Animal Models

8. Report species, strain, gender, age of animals and genetic modification status where applicable. Please detail housing and husbandry conditions and the source of animals.	Wwox null mice on FVB background. Animals were maintained in a SPF unit in a 12 h-light/dark cycle with ad libitum access to the food and water.
9. For experiments involving live vertebrates, include a statement of compliance with ethical regulations and identify the committee(s) approving the experiments.	All animal-related experiments were performed in accordance and with prior approval of the Hebrew University-Institutional Animal Care Use Committee (HU-IACUC).
10. We recommend consulting the ARRIVE guidelines (see link list at top right) (PLoS Biol. 8(6), e1000412, 2010) to ensure that other relevant aspects of animal studies are adequately reported. See author guidelines, under 'Reporting Guidelines'. See also: NIH (see link list at top right) and MRC (see link list at top right) recommendations. Please confirm compliance.	NA

E- Human Subjects

11. Identify the committee(s) approving the study protocol.	NA
12. Include a statement confirming that informed consent was obtained from all subjects and that the experiments conformed to the principles set out in the WMA Declaration of Helsinki and the Department of Health and Human Services Belmont Report.	NA
13. For publication of patient photos, include a statement confirming that consent to publish was obtained.	NA
14. Report any restrictions on the availability (and/or on the use) of human data or samples.	NA
15. Report the clinical trial registration number (at ClinicalTrials.gov or equivalent), where applicable.	NA
16. For phase II and III randomized controlled trials, please refer to the CONSORT flow diagram (see link list at top right) and submit the CONSORT checklist (see link list at top right) with your submission. See author guidelines, under 'Reporting Guidelines'. Please confirm you have submitted this list.	NA
17. For tumor marker prognostic studies, we recommend that you follow the REMARK reporting guidelines (see link list at top right). See author guidelines, under 'Reporting Guidelines'. Please confirm you have followed these guidelines.	NA

F- Data Accessibility

18: Provide a "Data Availability" section at the end of the Materials & Methods, listing the accession codes for data generated in this study and deposited in a public database (e.g. RNA-Seq data: Gene Expression Omnibus GSE39462, Proteomics data: PRIDE PXD000208 etc.) Please refer to our author guidelines for 'Data Deposition'.<br><br>Data deposition in a public repository is mandatory for:<br>a. Protein, DNA and RNA sequences<br>b. Macromolecular structures<br>c. Crystallographic data for small molecules<br>d. Functional genomics data<br>e. Proteomics and molecular interactions	The data is available as indicated in the Data availability section in the manuscript. Any additional data is available upon reasonable request.
19. Deposition is strongly recommended for any datasets that are central and integral to the study; please consider the journal's data policy. If no structured public repository exists for a given data type, we encourage the provision of datasets in the manuscript as a Supplementary Document (see author guidelines under 'Expanded View' or in unstructured repositories such as Dryad (see link list at top right) or Figshare (see link list at top right).	NA
20. Access to human clinical and genomic datasets should be provided with as few restrictions as possible while respecting ethical obligations to the patients and relevant medical and legal issues. If practically possible and compatible with the individual consent agreement used in the study, such data should be deposited in one of the major public access-controlled repositories such as dbGAP (see link list at top right) or EGA (see link list at top right).	NA
21. Computational models that are central and integral to a study should be shared without restrictions and provided in a machine-readable form. The relevant accession numbers or links should be provided. When possible, standardized format (SBML, CellML) should be used instead of scripts (e.g. MATLAB). Authors are strongly encouraged to follow the MIRIAM guidelines (see link list at top right) and deposit their model in a public database such as Biomodels (see link list at top right) or JWS Online (see link list at top right). If computer source code is provided with the paper, it should be deposited in a public repository or included in supplementary information.	NA

G- Dual use research of concern

22. Could your study fall under dual use research restrictions? Please check biosecurity documents (see link list at top right) and list of select agents and toxins (APHIS/CDC) (see link list at top right). According to our biosecurity guidelines, provide a statement only if it could.	NA
